# A Combined Gene Signature of Hypoxia and Notch Pathway in Human Glioblastoma and Its Prognostic Relevance

**DOI:** 10.1371/journal.pone.0118201

**Published:** 2015-03-03

**Authors:** Khushboo Irshad, Saroj Kant Mohapatra, Chitrangda Srivastava, Harshit Garg, Seema Mishra, Bhawana Dikshit, Chitra Sarkar, Deepak Gupta, Poodipedi Sarat Chandra, Parthaprasad Chattopadhyay, Subrata Sinha, Kunzang Chosdol

**Affiliations:** 1 Department of Biochemistry, All India Institute of Medical Sciences, New Delhi, India; 2 National Institute of Biomedical Genomics, Kalyani, West Bengal, India; 3 Department of Biochemistry, School of Life Science, University of Hyderabad, Hyderabad, India; 4 Department of Pathology, All India Institute of Medical Sciences, New Delhi, India; 5 Department of Neurosurgery, All India Institute of Medical Sciences, New Delhi, India; 6 National Brain Research Centre, Manesar, Gurgaon, Haryana, India; University Hospital of Navarra, SPAIN

## Abstract

Hypoxia is a hallmark of solid tumors including glioblastoma (GBM). Its synergism with Notch signaling promotes progression in different cancers. However, Notch signaling exhibits pleiotropic roles and the existing literature lacks a comprehensive understanding of its perturbations under hypoxia in GBM with respect to all components of the pathway. We identified the key molecular cluster(s) characteristic of the Notch pathway response in hypoxic GBM tumors and gliomaspheres. Expression of Notch and hypoxia genes was evaluated in primary human GBM tissues by q-PCR. Clustering and statistical analyses were applied to identify the combination of hypoxia markers correlated with upregulated Notch pathway components. We found well-segregated tumor—clusters representing high and low HIF-1α/PGK1-expressors which accounted for differential expression of Notch signaling genes. In combination, a five-hypoxia marker set (HIF-1α/PGK1/VEGF/CA9/OPN) was determined as the best predictor for induction of Notch1/Dll1/Hes1/Hes6/Hey1/Hey2. Similar Notch-axis genes were activated in gliomaspheres, but not monolayer cultures, under moderate/severe hypoxia (2%/0.2% O_2_). Preliminary evidence suggested inverse correlation between patient survival and increased expression of constituents of the hypoxia-Notch gene signature. Together, our findings delineated the Notch-axis maximally associated with hypoxia in resected GBM, which might be prognostically relevant. Its upregulation in hypoxia-exposed gliomaspheres signify them as a better *in-vitro* model for studying hypoxia-Notch interactions than monolayer cultures.

## Introduction

GBM is the most frequently diagnosed brain tumor with a high probability of aggressive relapse [1,2]. The median patient survival remains less than 2 years even with the use of aggressive chemotherapy and radiotherapy regimens [1,2,3]. Thus far, therapeutic strategies are largely guided by histological classification/analysis of glioma tumors. However, recently, gene expression profiling has been shown to correlate better with patient survival and prognosis [4]. Moreover, it identifies molecular signatures that regulate gliomagenesis and serve as targets for drug development [5,6].

As a rapidly growing tumor, GBM is characterized by central necrotic regions surrounded by hypoxic cancer cells. This niche confers resistance to therapy and harbors self-renewing cells responsible for tumor progression/recurrence [7]. Hypoxia essentially stabilizes HIF-1α (hypoxia inducible factor-1α), enabling transcriptional response that facilitates cell survival under hypoxic stress [8,9]. Genes like phosphoglycerate kinase 1 (PGK1), glucose transporter 1 (GLUT1), vascular endothelial growth factor (VEGF), erythropoietin (EPO) and carbonic anhydrase 9 (CA9) are directly regulated by HIF-1α and control glycolysis, angiogenesis, cell invasion/migration, etc. [8,9,10]. These, as well as osteopontin (OPN), are accredited tumor hypoxia markers and potential therapeutic targets [9,11,12,13].

Several cellular pathways crucial to the dynamics of tumorigenesis are deregulated by hypoxia [10]. Notch signaling is one such pathway with functions ranging from stem cell maintenance to induction of differentiation in embryonic and adult tissues [14,15]. Mammals have four Notch receptors (Notch1–4), five ligands (Delta-like (Dll) 1/3/4; Jagged (Jag) 1/2) and target genes belonging to Hes (Hes1/2/5/6) and Hey (Hey1/2) families of transcriptional repressors [14,15]. Although both oncogenic and tumor suppressive functions of Notch have been implicated depending on the tissue type [16], it mostly displays an oncogenic role in different malignancies including glioma [14,17,18].

Cooperative cross-talks between hypoxia and Notch signaling have previously been documented in mouse embryonic stem cells and progenitor cells [19,20,21,22], human breast cancer pathogenesis [17,23]; and maintenance of stem cells in different tumors [7,24,25,26,27,28,29,30,31]. Few studies have also addressed the altered expression of Notch signaling genes in surgically resected GBM [18,32,33] and their interaction with hypoxia in human [34,35] and rat glioma cell lines [36]. However, the studies in various tumors and cell lines have pointed to the correlation of different Notch components with diverse pathways, indicating pleiotropic roles of Notch under different conditions. Furthermore, the available genetic signatures of Notch/hypoxia pathways either contain few genes or are confined to a single phenomenon in tumorigenesis [33]. A generalized molecular signature related to both Notch and hypoxia; with potential implications in patient survival lacks in GBM. A combined marker from two pathways is likely to be a better predictor for prognosis. An in-depth analysis of association of hypoxia with an array of molecules comprising mammalian Notch receptors, ligands and target genes has not been reported in GBM until now. Also, an *in-vitro* model of hypoxia exposure mimicking the *in-vivo* Notch pathway activation is a requisite for further studies.

Therefore, our study aimed at characterizing the hypoxia-Notch pathway relationship; and identifying a molecular signature that might assist in GBM sub-grouping, thereby, aiding patient prognosis and adapted therapy. Towards this, we measured the quantitative expression of fifteen Notch and seven hypoxia signaling genes in surgically resected GBM tissues, to reveal the Notch pathway components whose activation is correlated with upregulation of hypoxia markers, either singly or in combination. The observed signature was correlated with GBM patient survival. *In-vitro* multicellular spheroids often provide an insight into cellular behaviour and regulation that differs in monolayer cultures [37]. For further understanding of hypoxia-Notch association, we assessed the similarities and differences of Notch response in GBM cell line monolayer and gliomasphere cultures under normoxia and moderate/severe hypoxia; and compared them with primary human GBM tumors.

## Materials and Methods

### Patients and tumor samples

Thirty-five surgically resected GBM samples were collected from Department of Neurosurgery/Neuropathology, All India Institute of Medical Sciences (AIIMS), New Delhi, after obtaining written consent from the patients. Ethical approval for the study was granted by Institute Ethics Committee, All India Institute of Medical Sciences, New Delhi (Ref. no.: IEC/NP-13/2011). The patients did not receive any specific therapy related to either hypoxia or Notch pathway. Histological diagnosis was done by Prof. Chitra Sarkar (Neuropathologist, AIIMS). For RNA isolation, tumor tissues were collected in RNA*later* solution (Ambion, USA) and stored at -70°C until further use. For immunohistochemistry, tissues were fixed in buffered 4% paraformaldehyde (pH 7.4) and embedded in paraffin. Among the patients, 14 were females and 21 were males. Normal human brain total RNA was purchased from Clontech (CA, USA) as control. Information on the survival status of 21 (60%) patients was obtained via telephonic conversation or by visiting the families. The cause of death was confirmed to be GBM.

### q-PCR

Total RNA from frozen tumors and cultured cell samples was extracted using TRI Reagent (Sigma-Aldrich, USA) and quantified using NanoDrop ND-1000 spectrophotometer (Thermo Fisher Scientific, USA). Contaminating genomic DNA was removed by DNase I (MBI Fermentas, Hanover, MD) treatment. Reverse transcriptase reactions were performed using random decamers (MWG, India) and reverse transcriptase enzyme (MBI Fermentas, Hanover, MD) in 20μl reaction volume. Real-time PCR was performed using Syto9 fluorescent dye (Invitrogen, Carlsbad, USA) in RotorGene 6000 Real Time PCR machine (Corbett Research, Australia). Primers for 7 hypoxia markers and 15 Notch pathway genes were designed using *Primer3* (http://frodo.wi.mit.edu/primer3/) and synthesized commercially (MWG, India) (S1 Table). The fold expression ratios of hypoxia markers and Notch genes were calculated relative to 18S rRNA internal control reference in case of GBM samples and normalized by the transcript levels of the genes found in normal brain sample. In *in-vitro* study, gene expression was calculated with respect to either one or multiple internal control references (18S rRNA, POLR2A and PPIA [38]; S2 Table) and normalized by the transcript levels found in normoxic monolayer cells. Relative fold change in gene expression was quantified by 2^-ΔΔC^_T_ method as follows.

$$Relative\;gene\;expression\;=\;\frac{2^{\left(Test\;sample\;reference\;gene\;C_{T}-\;Test\;sample\;target\;gene\;C_{T}\right)}}{2^{\left(Control\;sample\;reference\;gene\;C_{T}-Control\;sample\;target\;gene\;C_{T}\right)}}$$

When quantitation was done with respect to multiple reference genes, Relative Expression Software Tool (REST (http://www.gene-quantification.de/rest.html)) was used.

### Gene clustering and tertile analysis in GBM tertiles

Semi-supervised gene clustering was performed by arranging tumors in descending order of HIF-1α/PGK1/OPN/VEGF expression and grouping by tertiles. The mRNA expression ratios of hypoxia/Notch genes (normalized by normal brain transcript levels) were log-transformed (after replacing all zero-values by a small positive number, i.e., lower than the minimum non-zero data point) and subjected to clustering based on Spearman’s correlation and average linkage in Cluster 3.0. The output was visualized as heat map using Java TreeView. Differences in gene expression across high and low GBM tertiles were determined using Student’s *t*-test (1-tailed, unpaired).

### Principal components analysis (PCA) in GBM tertiles

Expression data were log-transformed, with base 2, after replacing all zero-values by a small positive number, i.e., half of the minimum non-zero data point. 2D and 3D plots were created using R (version 2.15.1) statistical software (available at http://www.r-project.org/) with the functions available in ‘stats’ package. Other attached packages were ‘gplots (version 2.11.0)’ and ‘rgl (version 0.92.894)’ using ‘Bioconductor version 2.11 (BiocInstaller 1.8.3)’. (see. zip folder; S2 File: R analysis supplemental content, which contains the R script master code and input data files for performing PCA).

**Construction of 2D PCA plots**. The GBMs were marked for HIF-1α/PGK1 expression using a colour code. A spectrum ranging from the brightest shade of red (for the highest HIF-1α/PGK1 expression) diminishing to lighter shades of pink; followed by light green intensifying to the darkest green (for the lowest HIF-1α/PGK1 expression) denoted the descending HIF-1α levels. The highest and lowest tertiles were identifiable as the 12 darkest red and 12 darkest green points, respectively.

**Construction of 3D PCA plots**. The GBMs were divided and coloured by tertiles as follows: Top 12 GBMs: red; 11 intermediate GBMs: yellow and bottom 12 GBMs: green. This colour scheme was followed for denoting HIF-1α/PGK1 expression. A 3D plot obtained after executing the commands in the R software console was rotated manually about the three axes or principal components to attain the best orientation projecting maximally separated high and low tertiles.

### Statistical analysis

Spearman’s rank correlation, sensitivity and specificity; and logistic regression were calculated using SPSS 11.5. In the tests, p-value of ≤ 0.05 was considered to be significant.

### Kaplan-Meier survival analysis

Kaplan-Meier survival analysis was done with R software using ‘survival’ package. Kaplan-Meier estimates were calculated based on the last follow-up time and the censor status of the samples and then plotted against the patient survival time. The setting of gene expression thresholds was done through visual inspection of the scatter plots between expression values and survival time. While for HIF-1α and Hes1 expression, the threshold was chosen to be 1.5-fold, OPN threshold expression was kept at 5.0-fold. For multiple genes, the combinatorial threshold was taken to be the arithmetic summation of the thresholds of individual genes i.e., HIF-1α + Hes1 + OPN = 1.5+1.5+5.0 = 8.0-fold. Difference between the survival curves was tested by the ‘survdiff’ function. P-value of < 0.05 was considered to be significant. (see. zip folder; S2 File: R analysis supplemental content, which contains the R script master code and input data file for performing Kaplan-Meier survival analysis).

### Cell culture and exposure to hypoxia for 24, 48 and 72 hours

GBM cell lines U87MG and U373MG were procured from American Type Tissue Culture while A172 was kindly provided by Prof. J.S. Castresana (Universidad de Navarra, Spain) [39]. Cells were maintained as described previously [40] and seeded in 25 cm^2^ vented tissue culture flasks (U87MG: 2.510^5^ cells/flask; A172: 4.010^5^ cells/flask; U373MG: 3.010^5^ cells/flask). On day 0, normoxia (20% O_2_) and severe hypoxia (0.2% O_2_) treatments were initiated in flasks showing 60–70% confluency using Anoxomat gas proportionater and chambers (Mart Microbiology, Netherlands). The cells were processed for RNA isolation/lysate preparation at 24/48/72 hours. Photomicrographs were taken using Eclipse-TE inverted phase contrast microscope (Nikon, Japan). All cell culture experiments were performed in 2–3 biological replicates.

### Gliomasphere formation assay

U87MG cells were seeded at a density of 0.5×10^5^ cells/well for monolayer adherent culture and 1.010^5^ cells/well for gliomasphere culture in 6-well plates using normal growth medium (DMEM with 10% FCS). On day 1, fresh normal medium was replaced in adherent culture wells while sphere culture wells were replaced with serum-free tumor sphere medium (DMEM F-12 with G5 and B27 growth factor supplements; Invitrogen, Carlsbad, USA). Different plates were maintained at normoxia (20% O_2_), moderate hypoxia (2% O_2_) and severe hypoxia (0.2% O_2_). The respective media were changed every alternate day. On day 10, the cells were processed for RNA isolation/lysate preparation. The same protocol was followed with U373MG cells for sphere formation.

For detailed methods, see S1 File Supplementary Data Text.

## Results

### Increased expression of Notch pathway genes correlates with the expression of hypoxia markers in GBM tumors

**Correlation of expression levels of individual genes**. mRNA expression of hypoxia markers and Notch genes was analyzed in 35 GBMs by q-PCR. Expression ≥ 1.5-fold relative to normal brain was considered as significant upregulation. Hypoxia markers were elevated in a high number of samples, indicating presence of hypoxia. PGK1, CA9, VEGF, OPN and HIF-1α were upregulated in 83%, 77%, 74%, 71% and 54% tumors, respectively (S3 Table). EPO and GLUT1, on the other hand, were upregulated in only 9% and 3% tumors, respectively. To rule out low input and confirm equal quantitation of samples having low target gene expression, 18S rRNA was quantified as an internal control reference.

Further corroboration for hypoxic status of the tumors was obtained by immunohistochemical analysis of HIF-1α and VEGF, where we found high protein levels in 17 randomly selected GBM samples with respect to normal brain, particularly in perinecrotic areas (S1 Fig). However, immunohistochemical analysis could only be done on different paraffin-embedded sections of the samples studied at mRNA level. The difference in the HIF-1α mRNA and protein expression in GBM 3, 5, 22 and 28 might, therefore, be attributable to intratumoral heterogeneity of hypoxia. Favourably enough, mRNA expression level is a measure that is quantitative and allowed us to correlate the expression of Notch genes with the degree of hypoxia present within a given region of tumor. The gradient of hypoxia is obtainable by quantifying mRNA levels of hypoxia markers. The same holds true for HIF-1α which, classically, is a hypoxia marker at the protein level. However, as only qualitative data is possible with immunohistochemistry, mRNA levels of HIF-1α were used in this study to determine the quantitative value for degree of hypoxia within a local tumor region.

Among Notch pathway genes, many were found to be overexpressed at mRNA level. Receptors Notch1, Notch2, Notch3; and ligands Dll1 and Jag1 were upregulated in 43%, 34%, 43%, 69% and 71% tumors, respectively. Among Notch target genes, Hes1, Hes6 and Hey1 were upregulated in 43%, 34% and 40% tumors, respectively. Other genes viz. Notch4, Dll3, Dll4, Jag2, Hes2, Hes5 and Hey2 were upregulated in ≤ 30% tumors (S3 Table).

Spearman’s correlation was calculated between the expression of hypoxia markers. HIF-1α, PGK1, VEGF and OPN showed significant positive correlations (p ≤ 0.04) with each other (Table 1). Notably, HIF-1α correlated with its known effector genes i.e., PGK1 (r = 0.747; p < 0.001), VEGF (r = 0.383; p = 0.012) and EPO (r = 0.331; p = 0.026) depicting its regulatory effect on their expression. With respect to Notch genes, of all the hypoxia markers, HIF-1α showed significant correlations (p ≤ 0.034) with the maximum number of Notch genes (14/15) by highest r values (Table 2); followed by PGK1 (13/15 Notch genes), OPN (10/15 Notch genes) and VEGF (8/15 Notch genes) (p ≤ 0.05). CA9 showed significant correlation with 1 gene (Hes2) only. Due to low expression, EPO and GLUT1 were not used for analyzing correlations.

**Table 1:** Spearman’s rank correlation coefficient and associated p-values between the expression of hypoxia markers with respect to each other.

Genes		HIF-1α	PGK1	VEGF	OPN	EPO	CA9
**HIF-1α**	Correlation Coefficient	1	.747(**)	.383(*)	.511(**)	.331(*)	0.056
	Sig. (1-tailed)	.	0	0.012	0.001	0.026	0.374
**VEGF**	Correlation Coefficient	.383(*)	.668(**)	1	.526(**)	.388(*)	.548(**)
	Sig. (1-tailed)	0.012	0	.	0.001	0.011	0
**OPN**	Correlation Coefficient	.511(**)	.597(**)	.526(**)	1	0.257	.303(*)
	Sig. (1-tailed)	0.001	0	0.001	.	0.068	0.038
**GLUT1**	Correlation Coefficient	.620(**)	.744(**)	.574(**)	.365(*)	.418(**)	.350(*)
	Sig. (1-tailed)	0	0	0	0.016	0.006	0.02
**EPO**	Correlation Coefficient	.331(*)	.299(*)	.388(*)	0.257	1	.342(*)
	Sig. (1-tailed)	0.026	0.04	0.011	0.068	.	0.022
**PGK1**	Correlation Coefficient	.747(**)	1	.668(**)	.597(**)	.299(*)	0.238
	Sig. (1-tailed)	0	.	0	0	0.04	0.084
**CA9**	Correlation Coefficient	0.056	0.238	.548(**)	.303(*)	.342(*)	1
	Sig. (1-tailed)	0.374	0.084	0	0.038	0.022	.

(*) Correlations found significant at the 0.05 level

(**) Correlations found significant at the 0.01 level

Abbreviations: Sig, significance

**Table 2:** Spearman’s rank correlation coefficient and associated p-values between the expression of Notch receptors, ligands, target genes and the hypoxia markers.

Genes		HIF-1α	PGK1	OPN	VEGF	CA9
**Notch1**	Correlation Coefficient	.566(**)	.450(**)	.296(*)	.293(*)	0.173
	Sig. (1-tailed)	0	0.003	0.042	0.044	0.161
**Notch2**	Correlation Coefficient	.434(**)	.420(**)	.322(*)	0.222	0.19
	Sig. (1-tailed)	0.005	0.006	0.03	0.1	0.138
**Notch3**	Correlation Coefficient	.312(*)	0.235	0.136	0.054	-0.002
	Sig. (1-tailed)	0.034	0.087	0.218	0.379	0.495
**Notch4**	Correlation Coefficient	.494(**)	.373(*)	.378(*)	.400(**)	0.121
	Sig. (1-tailed)	0.001	0.014	0.013	0.009	0.245
**Dll1**	Correlation Coefficient	.568(**)	.539(**)	.286(*)	0.213	0.069
	Sig. (1-tailed)	0	0	0.048	0.109	0.348
**Dll3**	Correlation Coefficient	.551(**)	.499(**)	.332(*)	0.249	0.034
	Sig. (1-tailed)	0	0.001	0.026	0.074	0.423
**Dll4**	Correlation Coefficient	.455(**)	.452(**)	0.255	.476(**)	0.196
	Sig. (1-tailed)	0.003	0.003	0.07	0.002	0.13
**Jag1**	Correlation Coefficient	.402(**)	.293(*)	.288(*)	0.23	0.232
	Sig. (1-tailed)	0.008	0.044	0.047	0.092	0.09
**Jag2**	Correlation Coefficient	.366(*)	.480(**)	.285(*)	.377(*)	-0.068
	Sig. (1-tailed)	0.015	0.002	0.049	0.013	0.348
**Hes1**	Correlation Coefficient	.599(**)	.668(**)	.572(**)	.471(**)	-0.147
	Sig. (1-tailed)	0	0	0	0.002	0.199
**Hes2**	Correlation Coefficient	0.113	0.209	0.154	.368(*)	.402(**)
	Sig. (1-tailed)	0.258	0.114	0.188	0.015	0.008
**Hes5**	Correlation Coefficient	.506(**)	.361(*)	0.2	0.114	-0.032
	Sig. (1-tailed)	0.001	0.017	0.125	0.258	0.428
**Hes6**	Correlation Coefficient	.573(**)	.561(**)	.518(**)	.300(*)	0.039
	Sig. (1-tailed)	0	0	0.001	0.04	0.412
**Hey1**	Correlation Coefficient	.790(**)	.790(**)	.459(**)	.499(**)	0.088
	Sig. (1-tailed)	0	0	0.003	0.001	0.309
**Hey2**	Correlation Coefficient	.640(**)	.518(**)	0.213	0.257	-0.251
	Sig. (1-tailed)	0	0.001	0.109	0.068	0.073

(*) Correlations found significant at the 0.05 level

(**) Correlations found significant at the 0.01 level

Abbreviations: Sig, significance

**Fig 1:**
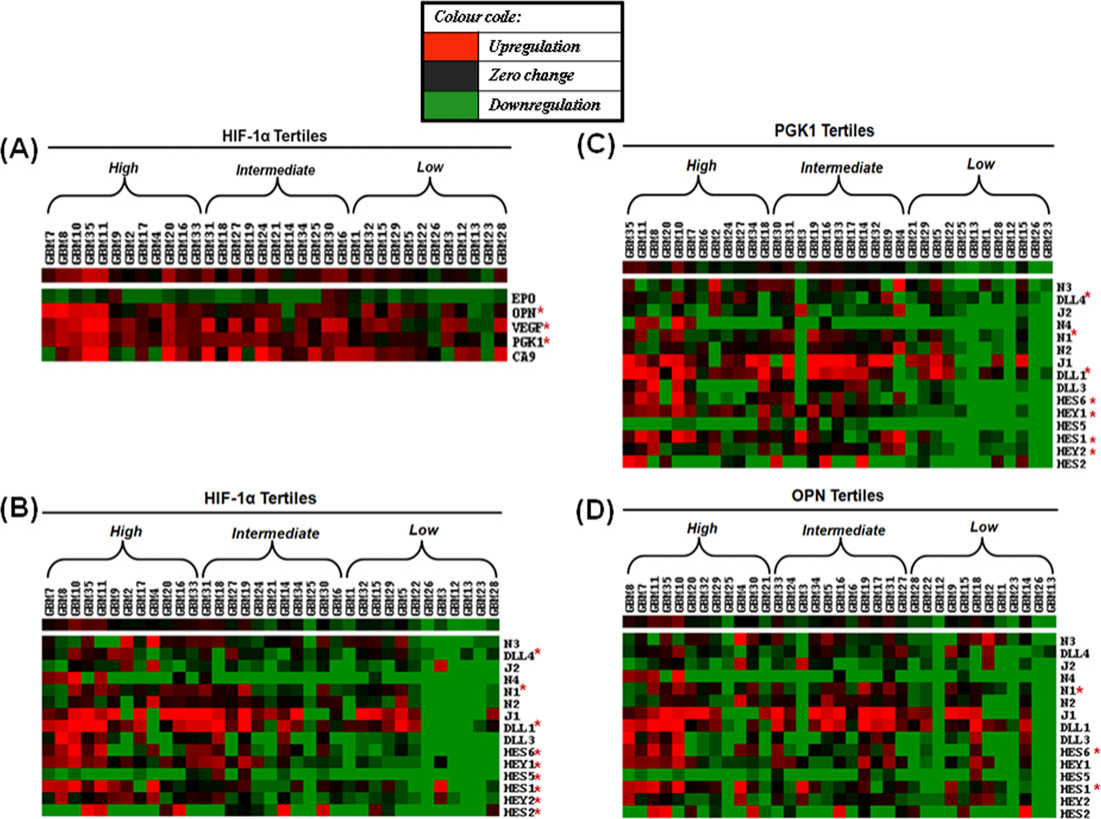
Heat maps showing gene cluster analysis in GBM tumor tertiles. **(A)** Clustering of hypoxia genes in GBMs sorted in decreasing order of HIF-1α expression. PGK1, VEGF and OPN displayed greater upregulation in the high HIF-1α GBM tertile as compared to the low tertile. Clustering of Notch signaling genes in GBMs sorted in decreasing order of **(B)** HIF-1α expression **(C)** PGK1 expression and **(D)** OPN expression showed maximum number of Notch signaling genes (9/15) to be upregulated in the high HIF-1α GBM tertile, followed by high PGK1 and OPN tertiles which exhibited increased expression of 7/15 and 3/15 Notch genes, respectively. Gene expression found significantly different across the high and low tertiles (p ≤ 0.05) has been indicated by an asterisk (*).

**Heat map of Notch genes based on the expression of individual hypoxia markers—comparison of tertiles**. In order to find out whether the degree of hypoxia has correlation with the expression of individual or collective Notch genes, we arranged the tumors in decreasing order of HIF-1α expression and grouped them by tertiles as: high (≥ 3.6-fold; 12 tumors); intermediate (3.5 to 0.8-fold; 11 tumors) and low (≤ 0.8-fold; 12 tumors) HIF-1α GBM tertiles. The purpose of forming tertiles was to study in-group comparison of differential expression of Notch genes across the two ends of a tumor population which showed the maximum or minimum expression of hypoxia marker. Keeping the fixed order, a heat map was generated. We first analyzed hypoxia markers PGK1, OPN and VEGF in HIF-1α GBM tertiles to visualize their expression in relation to HIF-1α expression. They revealed greater upregulation in the high HIF-1α tertile than low tertile with a significant difference (p ≤ 0.05) (Fig. 1(A)), confirming their association with HIF-1α. We then proceeded to examine the Notch genes likewise for their association with the expression level of all hypoxia markers. 9/15 Notch genes (Notch1, Dll1, Dll4, Hes1, Hes2, Hes5, Hes6, Hey1 and Hey2) displayed greater upregulation in the high HIF-1α tertile compared to the low tertile, with a significant difference (p ≤ 0.05) (Fig. 1(B)). In high PGK1 and high OPN tertiles, 7/15 (Notch1, Dll1, Dll4, Hes1, Hes6, Hey1, Hey2) and 3/15 (Notch1, Hes1, Hes6) Notch genes were observed to be increased, respectively (Fig. 1(C, D)). Detailed analysis is shown in S4–S7 Tables. Thus, our results imply a positive correlation between the expression of Notch pathway genes and hypoxia markers in GBM. However, with VEGF and CA9, the clustering pattern of Notch genes was relatively diffuse (S2 Fig; S8 and S9 Tables).

### Principal components analysis (PCA) of the differential expression of Notch genes extracts well-segregated clusters of high and low HIF-1α and PGK1 expressing GBM tertiles

**Fig 2:**
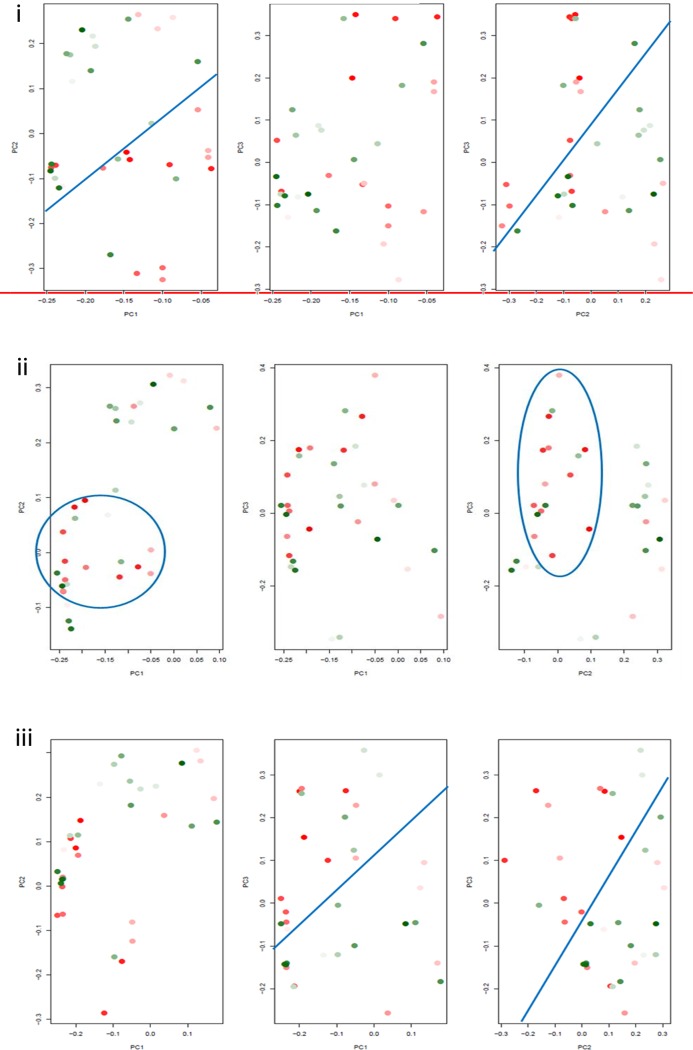
2D PCA plots showing clustering of high and low HIF-1α expressing GBM tertiles on the basis of differential expression of Notch pathway genes. Each data point in a plot denotes a GBM and depicts the score created by weighted summation of Notch genes’ expression in that tumor. The colour gradient from darkest red to darkest green denotes the highest HIF-1α expression to the lowest HIF-1α expression in the data points representing tumors. The demarcation between high HIF-1α GBM tertile and low HIF-1α GBM tertile in each plot has been displayed by using either lines or curves (blue coloured) which have been drawn manually to reveal the closely spaced cluster. Cluster formation was assessed by counting the number of the darkest red and the darkest green data points on both sides of the line; or those enclosed within the curve. GBM scores were created by the combination of either **(i)** all 15 Notch genes or **(ii)** 9 Notch genes (Notch1, Dll1, Dll4, Hes1, Hes2, Hes5, Hes6, Hey1, Hey2) and **(iii)** 7 Notch genes (Notch1, Dll1, Hes1, Hes2, Hes6, Hey1, Hey2) selected on the basis of HIF-1α heat map. HIF-1α-ordering of the tumors produced well-separated clusters of high and low tertiles based on 15 Notch genes’ as well as 7 Notch genes’ expression.

**Fig 3:**
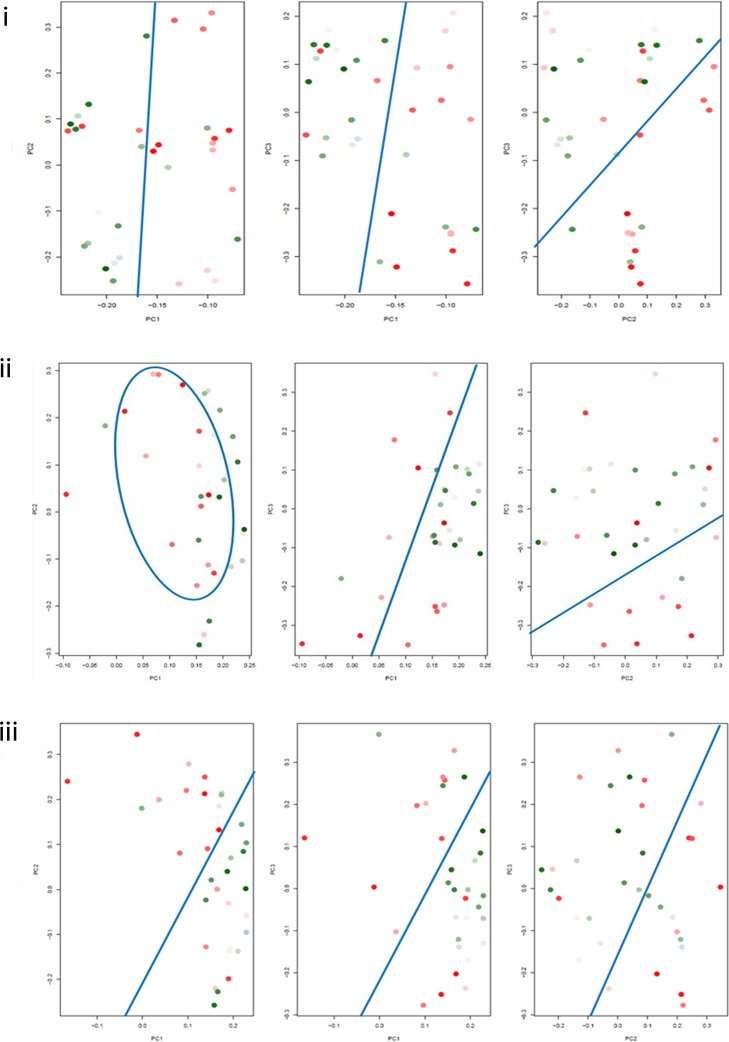
2D PCA plots showing clustering of high and low PGK1 expressing GBM tertiles on the basis of differential expression of Notch pathway genes. Each data point in a plot denotes a GBM and depicts the score created by weighted summation of Notch genes’ expression in that tumor. The colour gradient from darkest red to darkest green denotes the highest PGK1 expression to the lowest PGK1 expression in the data points representing tumors. The demarcation between high PGK1 GBM tertile and low PGK1 GBM tertile in each plot has been displayed by using either lines or curves (blue coloured) which have been drawn manually to reveal the closely spaced cluster. Cluster formation was assessed by counting the number of the darkest red and the darkest green data points on both sides of the line; or those enclosed within the curve. GBM scores were created by the combination of either **(i)** all 15 Notch genes or **(ii)** 7 Notch genes (Notch1, Dll1, Dll4, Hes1, Hes6, Hey1, Hey2) and **(iii)** 6 Notch genes (Notch1, Dll1, Hes1, Hes6, Hey1, Hey2) selected on the basis of PGK1 heat map. Similar to the case of HIF-1α, PGK1-ordering of the tumors also generated well-separated clusters of high and low tertiles based on 15 Notch genes’ expression.

**Fig 4:**
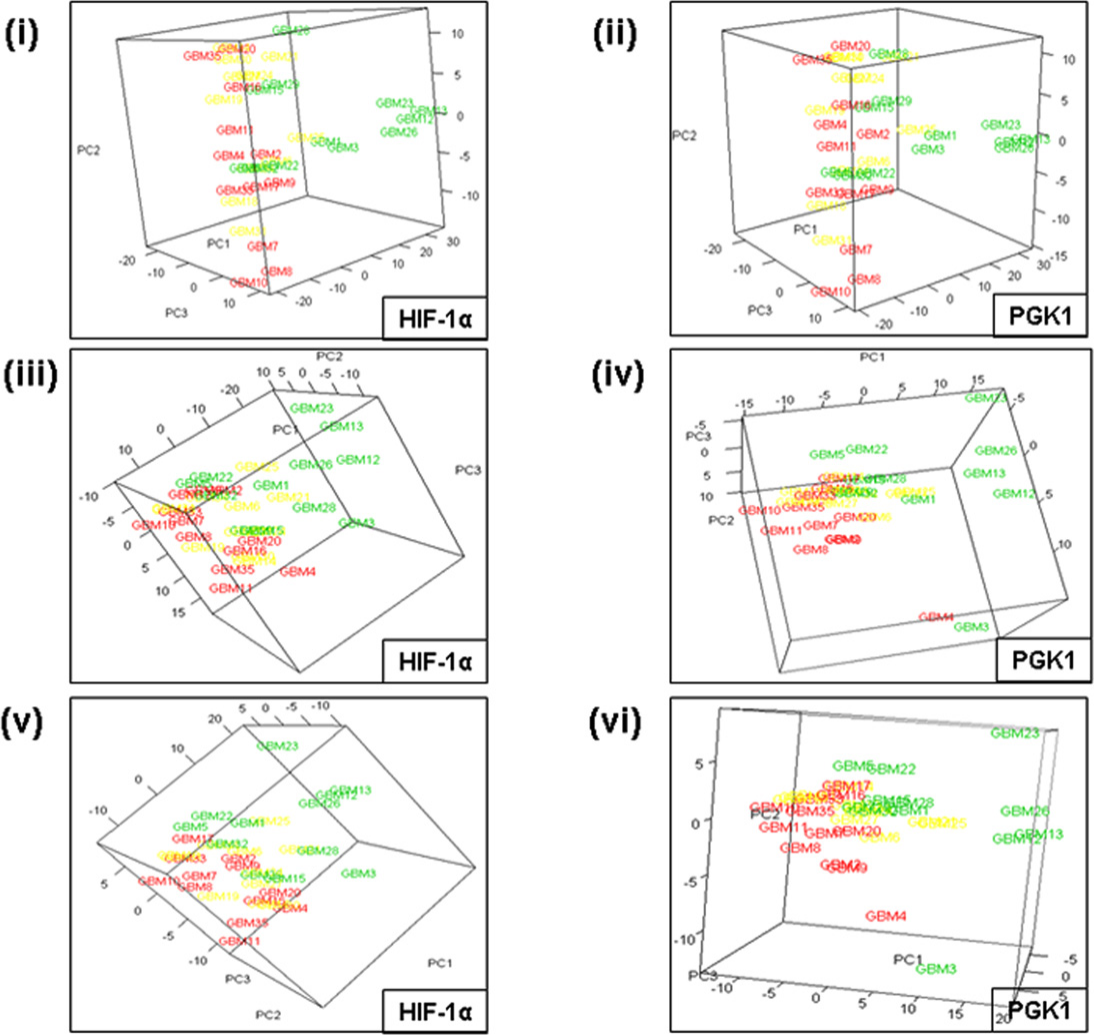
3D PCA plots showing clustering of GBM tumors belonging to HIF-1α tertiles and PGK1 tertiles on the basis of differential expression of Notch pathway genes. Colouring scheme in 3D plots: GBMs have been coloured as red data points for high tertile (12 tumors), yellow data points for intermediate tertile (11 tumors) and green data points for low tertile (12 tumors), respectively. Similar to 2D plots, the combinations of Notch genes used to create the principal components in each 3D plot were as follows: **(i & ii)** all 15 Notch genes; **(iii)** 9 genes (Notch1, Dll1, Dll4, Hes1, Hes2, Hes5, Hes6, Hey1, Hey2); **(iv)** 7 genes (Notch1, Dll1, Dll4, Hes1, Hes6, Hey1, Hey2); **(v)** 7 genes (Notch1, Dll1, Hes1, Hes2, Hes6, Hey1, Hey2); **(vi)** 6 genes (Notch1, Dll1, Hes1, Hes6, Hey1, Hey2). Both HIF-1α- and PGK1-ordering of the tumors produced well-separated clusters of high and low tertiles based on 15 or selected Notch genes’ expression.

PCA was employed as a converse modality to validate gene clustering results i.e., clustering of tumors based on collective Notch genes’ expression. We tested if the GBMs, pre-structured by decreasing hypoxia marker expression, would form discrete clusters accounting for the differential expression of Notch pathway genes. We used the two hypoxia markers, HIF-1α and PGK1, which showed the maximum association with expression of Notch genes in the heat maps. In a PCA plot, each data point or GBM score was created by weighted summation of Notch genes’ expression. The aim was to determine the best ordering of GBMs leading to best visualization of separated high and low tertiles. The HIF-1α/PGK1 levels in GBMs were denoted by colour codes as described in Figs. 2, 3 and 4.

**2D plots**. Using summation of all 15 Notch genes, segregation of high and low HIF-1α/PGK1 tertiles was observed but with intermixing of few GBMs on either sides (Figs. 2-i; 3-i). When only the significantly associated Notch genes were selected from HIF-1α (9 genes—Notch1, Dll1, Dll4, Hes1, Hes2, Hes5, Hes6, Hey1, Hey2) and PGK1 (7 genes—Notch1, Dll1, Dll4, Hes1, Hes6, Hey1, Hey2) heat maps (Fig. 1(B, C)), the separation was not as discernible as with 15 genes in both HIF-1α and PGK1 2D plots (Figs. 2-ii; 3-ii). Upon excluding Dll4 and Hes5 from the summation in case of HIF-1α plot; and Dll4 in case of PGK1 plot; considering their overexpression in very less tumors (S3 Table), HIF-1α-ordering led to better tertile separation with the least intermixing of red and green data points on either side (Fig. 2-iii) while PGK1-ordering showed less improvement (Fig. 3-iii). Hence, both HIF-1α- and PGK1-ordering produced distinct GBM clusters in 2D plots based on combined expression of Notch1, Dll1, Hes1, Hes2, Hes6, Hey1 and Hey2 (Hes2 inclusive only in case of HIF-1α-ordering).

**3D plots**. Using 15 Notch genes, both HIF-1α and PGK1-ordering led to well-demarcated clusters of high and low tertile GBMs (Fig. 4-i, ii) in 3D plots. With selected Notch genes also, HIF-1α- and PGK1-sorting captured tightly clustered pattern of tertiles (Fig. 4-iii, iv, v, vi), except for two data points in case of PGK1.

Overall, both the modalities of 2D and 3D PCA plots supported the effectiveness of HIF-1α and PGK1 expression to define tumor sub-grouping based on differential expression of Notch genes.

### Expression of single hypoxia markers diagnoses increased expression of Notch genes

Sensitivity and specificity analysis was applied to test each hypoxia marker as a potential predictor for overexpression of Notch genes in the 35 GBM dataset. Overexpression of Notch genes was taken to be the value greater than the median expression of the hypoxia marker in question. Sensitivity/specificity ≥ 50% were taken as the cut-offs for estimating the diagnostic accuracy of a predictor. Among the hypoxia markers analyzed, HIF-1α expression was found as diagnostic for the overexpression of maximum number of Notch genes viz. Notch1, Dll1, Jag1, Hes1, Hes6 and Hey1 (Table 3). PGK1 and OPN also displayed association with the overexpression of two genes i.e., Dll1 and Jag1. However, CA9 was associated with only Jag1 while VEGF associated with none (S10 Table).

**Table 3:** Summary of sensitivity and specificity of HIF-1α, PGK1 and OPN expression in diagnosis of Notch genes’ overexpression in 35 GBM tumors.

Sensitivity and specificity of HIF-1α expression:							
Variable (transformed on basis of HIF-1α)	Count			PPV (%)	Sensitivity (%)	NPV (%)	Specificity (%)
		HIF-1α (predictor)					
		1	0				
Notch1	1	9	2	81.8	**52.9**	66.7	**88.9**
Notch2	1	7	3	70.0	41.2	60.0	**83.3**
Notch3	1	8	6	57.1	47.1	57.1	**66.7**
Notch4	1	4	0	100.0	23.5	58.1	**100.0**
Dll1	1	15	8	65.2	**88.2**	83.3	**55.6**
Dll3	1	5	1	83.3	29.4	58.6	**94.4**
Dll4	1	6	1	85.7	35.3	60.7	**94.4**
Jag1	1	15	9	62.5	**88.2**	81.8	**50.0**
Jag2	1	2	1	66.7	11.8	53.1	**94.4**
Hes1	1	9	4	69.2	**52.9**	63.6	**77.8**
Hes2	1	4	4	50.0	23.5	51.9	**77.8**
Hes5	1	2	0	100.0	11.8	54.5	**100.0**
Hes6	1	10	2	83.3	**58.8**	69.6	**88.9**
Hey1	1	12	0	100.0	**70.6**	78.3	**100.0**
Hey2	1	7	1	87.5	41.2	63.0	**94.4**

Sensitivity and specificity of PGK1 expression:							
Variable (transformed on basis of PGK1)	Count			PPV (%)	Sensitivity (%)	NPV (%)	Specificity (%)
		PGK1 (predictor)					
		1	0				
Notch1	1	4	0	100.0	22.2	54.8	**100.0**
Notch2	1	0	2	0	0	45.5	**88.2**
Notch3	1	1	2	33.3	5.6	46.9	**88.2**
Notch4	1	4	0	100.0	22.2	54.8	**100.0**
Dll1	1	10	5	66.7	**55.6**	60.0	**70.6**
Dll3	1	4	1	80.0	22.2	53.3	**94.1**
Dll4	1	2	1	66.7	11.1	50.0	**94.1**
Jag1	1	10	8	55.6	**55.6**	52.9	**52.9**
Jag2	1	1	1	50.0	5.6	48.5	**94.1**
Hes1	1	5	2	71.4	27.8	53.6	**88.2**
Hes2	1	4	1	80.0	22.2	53.3	**94.1**
Hes5	1					48.6	**100.0**
Hes6	1	5	0	100.0	27.8	56.7	**100.0**
Hey1	1	8	0	100.0	44.4	63.0	**100.0**
Hey2	1	2	1	66.7	11.1	50.0	**94.1**

Sensitivity and specificity of OPN expression:							
Variable (transformed on basis of OPN)	Count			PPV (%)	Sensitivity (%)	NPV (%)	Specificity (%)
		OPN (predictor)					
		1	0				
Notch1	1	2	2	50.0	11.1	48.4	**88.2**
Notch2	1	2	0	100.0	11.1	51.5	**100.0**
Notch3	1	2	1	66.7	11.1	50.0	**94.1**
Notch4	1	4	0	100.0	22.2	54.8	**100.0**
Dll1	1	10	7	58.8	**55.6**	55.6	**58.8**
Dll3	1	3	3	50.0	16.7	48.3	**82.4**
Dll4	1	1	3	25.0	5.6	45.2	**82.4**
Jag1	1	13	8	61.9	**72.2**	64.3	**52.9**
Jag2	1	2	1	66.7	11.1	50.0	**94.1**
Hes1	1	6	1	85.7	33.3	57.1	**94.1**
Hes2	1	4	1	80.0	22.2	53.3	**94.1**
Hes5	1	0	1	0	0	47.1	**94.1**
Hes6	1	5	1	83.3	27.8	55.2	**94.1**
Hey1	1	7	3	70.0	38.9	56.0	**82.4**
Hey2	1	2	1	66.7	11.1	50.0	**94.1**

Note: Gene expression values were transformed into binary data where 1 denotes values ≥ HIF-1α or PGK1 or OPN median expression and 0 denotes values ≤ HIF-1α or PGK1 or OPN median expression. Sensitivity and specificity ≥ 50% were taken as cut-offs for predictive value of HIF-1α or PGK1 or OPN and have been indicated in bold.

Abbreviations: PPV, positive predictive value; NPV, negative predictive value

### A combination of five hypoxia markers (HIF-1α, PGK1, VEGF, CA9 and OPN) is the best predictor for Notch upregulation in GBM tumors

We analyzed different logistic regression models in order to evaluate the strength of association between a possible predictor or combination of predictors i.e., hypoxia marker(s); and increased Notch genes’ expression in GBMs.

Individually, HIF-1α was observed as the best predictor among all hypoxia markers, associated by high r values with the overexpression of maximum number of Notch genes, namely Notch4, Dll1, Dll3, Hes1, Hes6 and Hey1 (Table 4). HIF-1α was followed by OPN, PGK1 and VEGF which associated with 5 (Dll3, Hes1, Hes6, Hey1 and Hey2), 4 (Jag1, Hes2, Hes6 and Hey1) and 3 (Jag1, Hes2 and Hes6) genes, respectively. CA9 associated with only Jag1.

**Table 4:** Summary of results of linear regression analysis with each single hypoxia marker used as a regressor for its association with expression of individual Notch genes.

Predictor: HIF-1α				
Notch signaling gene (Dependent variable)	r	r^2^	p-value of regression model	β
Notch4	0.50	0.21	0.005	0.52
Dll1	0.40	0.16	0.02	1.29
Dll3	0.51	0.26	0.002	0.46
Hes1	0.44	0.19	0.009	0.42
Hes6	0.47	0.22	0.005	0.30
Hey1	0.49	0.24	0.003	0.18

Predictor: OPN				
Notch signaling gene (Dependent variable)	r	r^2^	p-value of regression model	β
Dll3	0.79	0.62	0.00	0.18
Hes1	0.39	0.15	0.02	0.10
Hes6	0.46	0.21	0.006	0.07
Hey1	0.41	0.17	0.01	0.04
Hey2	0.52	0.27	0.001	0.03

Predictor: PGK1				
Notch signaling gene (Dependent variable)	r	r^2^	p-value of regression model	β
Jag1	0.39	0.15	0.02	3.37
Hes2	0.34	0.12	0.045	0.29
Hes6	0.44	0.19	0.008	0.19
Hey1	0.35	0.12	0.04	0.09

Predictor: VEGF				
Notch signaling gene (Dependent variable)	r	r^2^	p-value of regression model	β
Jag1	0.39	0.16	0.02	0.94
Hes2	0.36	0.13	0.03	0.08
Hes6	0.39	0.15	0.02	0.05

Predictor: CA9				
Notch signaling gene (Dependent variable)	r	r^2^	p-value of regression model	β
Jag1	0.48	0.23	0.004	0.14

Note: Only the Notch genes found to be significantly associated (p ≤ 0.05) with the predictor of regression model have been shown in case of each predictor.

Abbreviations: r, correlation coefficient; r^2^, coefficient of determination; β, regression coefficient

In combination, a five-hypoxia marker set containing HIF-1α, PGK1, VEGF, CA9 and OPN was found to be the best predictor of overexpression of Dll1, Dll3, Hes1, Hes6, Hey1 and Hey2 (Table 5), among the 14 possible combinations of markers analyzed (S1 File Supplementary Data Text; S11 Table). This combination acted as a better predictor than HIF-1α alone since it associated with the same Notch subset (Dll1, Dll3, Hes1, Hes6 and Hey1) by higher r values (Tables 4 and 5), hence, proving to be better than any other combination or single marker studied.

**Table 5:** Summary of results of linear regression analysis using a combination of five hypoxia markers (HIF-1α, PGK1, VEGF, CA9 and OPN) as independent variables for their combined association with the expression level of individual Notch genes.

Predictor: HIF-1α, PGK1, VEGF, CA9, OPN					
Notch signaling gene (Dependent variable)	r	r^2^	p-value of regression model	β	
Dll1	0.58	0.34	0.029	HIF-1α	2.21
				PGK1	3.21
				VEGF	-0.37
				CA9	-0.04
				OPN	-0.73
Dll3	0.90	0.80	0.000	HIF-1α	-0.46
				PGK1	0.12
				VEGF	-0.07
				CA9	-0.004
				OPN	0.29
Hes1	0.57	0.32	0.037	HIF-1α	0.44
				PGK1	0.60
				VEGF	-0.04
				CA9	-0.01
				OPN	-0.09
Hes6	0.66	0.44	0.004	HIF-1α	0.27
				PGK1	0.38
				VEGF	-0.005
				CA9	-0.008
				OPN	-0.05
Hey1	0.58	0.34	0.030	HIF-1α	0.21
				PGK1	0.28
				VEGF	-0.03
				CA9	-0.003
				OPN	-0.05
Hey2	0.67	0.44	0.003	HIF-1α	-0.09
				PGK1	0.08
				VEGF	-0.02
				CA9	-0.001
				OPN	0.04

Note: Only the Notch genes found to be significantly associated (p < 0.05) with the predictor of regression model have been shown.

Abbreviations: r, correlation coefficient; r^2^, coefficient of determination; β, regression coefficient

Taken together, all statistical tools pointed to HIF-1α as the best single hypoxia marker to identify upregulated Notch signaling, followed by PGK1, OPN and VEGF (Table 6). However, logistic regression, an analysis that facilitated assessment of multiple predictors simultaneously for association with each Notch gene, substantiated the combination of HIF-1α, PGK1, VEGF, CA9 and OPN hypoxia markers as best correlated with the upregulation of Notch pathway components (Table 5).

**Table 6:** Summary of each computational/statistical analysis listing the significant associations of hypoxia markers with the expression of Notch signaling genes.

Type of analysis	Notch genes associated significantly with the hypoxia markers	
	*Hypoxia marker*	*Notch signaling genes*
Spearman’s correlation analysis	HIF-1α	*14 genes*- Notch1, Notch2, Notch3, Notch4, Dll1, Dll3, Dll4, Jag1, Jag2, Hes1, Hes5, Hes6, Hey1, Hey2
	PGK1	*13 genes*- Notch1, Notch2, Notch4, Dll1, Dll3, Dll4, Jag1, Jag2, Hes1, Hes5, Hes6, Hey1, Hey2
	OPN	*10 genes*- Notch1, Notch2, Notch4, Dll1, Dll3, Jag1, Jag2, Hes1, Hes6, Hey1
	VEGF	*8 genes*- Notch1, Notch4, Dll4, Jag2, Hes1, Hes2, Hes6, Hey1
	CA9	*1 gene-* Hes2
Gene clustering analysis (Heat maps)	HIF-1α	*9 genes*- Notch1, Dll1, Dll4, Hes1, Hes2, Hes5, Hes6, Hey1, Hey2
	PGK1	*7 genes*- Notch1, Dll1, Dll4, Hes1, Hes6, Hey1, Hey2
	OPN	*3 genes*- Notch1, Hes1, Hes6
	VEGF	*1 gene-* Dll4
	CA9	*1 gene-* Hey2
Principal components analysis	HIF-1α	*7 genes*- Notch1, Dll1, Hes1, Hes2, Hes6, Hey1, Hey2
	PGK1	*6 genes*- Notch1, Dll1, Hes1, Hes6, Hey1, Hey2
Sensitivity and specificity analysis	HIF-1α	*6 genes*- Notch1, Dll1, Jag1, Hes1, Hes6, Hey1
	PGK1	*2 genes*- Dll1, Jag1
	OPN	*2 genes*- Dll1, Jag1
	CA9	*1 gene-* Jag1
Logistic regression analysis (calculated using single hypoxia marker at a time)	HIF-1α	*6 genes*- Notch4, Dll1, Dll3, Hes1, Hes6, Hey1
	OPN	*5 genes*- Dll3, Hes1, Hes6, Hey1, Hey2
	PGK1	*4 genes*- Jag1, Hes2, Hes6, Hey1
	VEGF	*3 genes*- Jag1, Hes2, Hes6
	CA9	*1 gene-* Jag1

### A combination of six Notch pathway genes (Notch1, Dll1, Hes1, Hes6, Hey1 and Hey2) is most associated with the expression of hypoxia markers

Results from all cross validation tests were compared to arrive at the Notch subset which showed the strongest association with the hypoxia markers belonging to the five-marker set (Table 6). Notch1, Dll1, Hes1, Hes6, Hey1 and Hey2 were inferred to be the genes most associated with the expression of 3 hypoxia markers (HIF-1α, PGK1 and OPN) out of the five-marker panel in the GBM tumors studied. In case of each hypoxia marker, we considered those Notch genes that were significantly associated with it by at least 2 out of 6 statistical analyses.

### Poor patient survival correlates with increased expression of the components of hypoxia-Notch signaling axis

**Fig 5:**
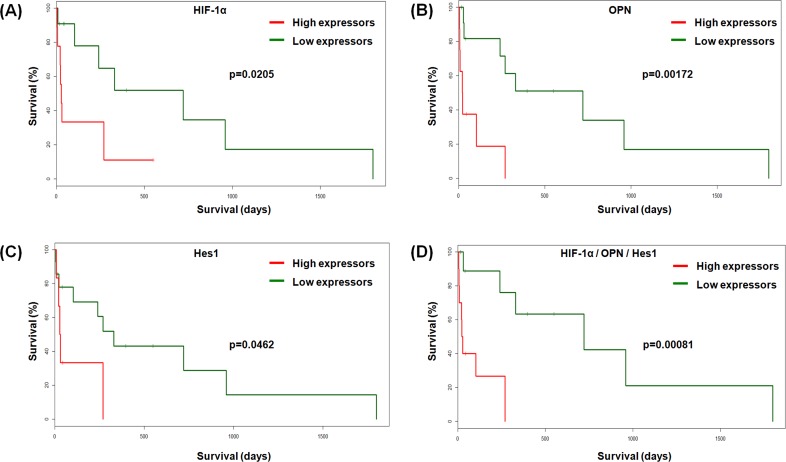
Survival analysis of GBM patients in relation to expression of the components of hypoxia-Notch gene signature. Kaplan-Meier plots showing comparison of survival of GBM patients (n = 21) segregated as high and low expressors of HIF-1α, OPN and Hes1, individually as well as in combination. The p-value indicates the significance of the difference in survival between the two groups of GBM samples. The fold expression thresholds that were used to draw the plots were: HIF-1α = 1.5; OPN = 5.0; Hes1 = 1.5 and HIF-1α/OPN/Hes1 = 8.0.

Because of difficulties in follow-up record maintenance in the institute we could obtain patient survival data in only 21 cases out of 35. Kaplan-Meier survival curves were plotted using the expression of the identified hypoxia-Notch subset genes (S12 Table). Of the genes analyzed, HIF-1α, OPN and Hes1 expression, individually, displayed an inverse relationship with patient survival (p = 0.0205, p = 0.00172 and p = 0.0462, respectively). Interestingly, the poor survival outcome was more significantly correlated with the combined upregulation of HIF-1α/OPN/Hes1 (p = 0.00081) (Fig. 5(A-D)).

### Gliomaspheres mimic the hypoxia-activated Notch signaling axis of primary GBM tumors

We first analyzed the expression of hypoxia markers and Notch genes in monolayer cultures of different GBM cell lines (U87MG, A172 and U373MG) grown in normal DMEM and exposed to severe hypoxia (0.2% O_2_) for 24, 48 and 72 hours. Increase in CA9, PGK1, VEGF and GLUT1 in all cell lines demonstrated their hypoxia-induced upregulation (Table 7; S3A Fig). HIF-1α protein was found to be elevated in hypoxia-exposed U373MG cells but showed an anomalous response in U87MG cells exposed to severe hypoxia with respect to normoxia (S3A Fig). This, however, has not been analyzed further, but could be contributed by HIF-1α protein modulation by p53 [41,42,43]. With respect to Notch signaling genes, the different monolayer cell lines displayed differential response to hypoxia at the studied time points (Table 7). Notch3 and Jag2 were found to be upregulated in all three cell lines studied. But Jag1, Dll4, Hey1, Hes1and Hes2 were increased selectively in one or two of the cell lines (Table 7; S3A Fig). Thus, GBM monolayer cells cultured under hypoxia exhibited a limited Notch response confined to few genes, which differed from that observed in primary tumors. The extent of gene upregulation was also noted to be lesser in monolayer cultures. This could be because each of the established cell lines represents one kind of genetic aberrations unlike solid tumors and also due to the possible role of culture conditions.

**Table 7:** mRNA fold expression ratios of hypoxia markers and Notch signaling genes in monolayer cultures of GBM cell lines (U87MG, A172 and U373MG) exposed to 0.2% O_2_ for 24, 48 and 72 hours.

Genes	U87MG (0.2% O_2_ vs 20% O_2_)			A172 (0.2% O_2_ vs 20% O_2_)			U373MG (0.2% O_2_ vs 20% O_2_)		
	24 hours	48 hours	72 hours	24 hours	48 hours	72 hours	24 hours	48 hours	72 hours
HYPOXIA MARKERS:									
HIF-1α	1.0	1.1	0.9	0.6	0.2	0.4	0.9	0.8	0.7
CA9	**1097.0**	**1632.0**	**1121.0**	**1071.0**	**634.0**	**8009.0**	**317.4**	**675.6**	**445.7**
PGK1	**9.8**	**26.1**	**35.0**	**3.6**	**6.7**	**15.8**	0.7	**5.3**	**10.6**
GLUT1	**62.0**	**38.0**	**56.0**	**3.0**	**4.0**	**3.5**	0.5	**2.6**	1.5
VEGF	**6.3**	**10.6**	**23.9**	**2.5**	**4.4**	**7.1**	**12.0**	**15.6**	**19.7**
OPN	1.2	1.4	**3.2**	0.5	0.2	0.1	Not detectable		
EPO	Not detectable			**1.8**	0.9	1.0	Not detectable		
NOTCH SIGNALING GENES:									
NOTCH RECEPTORS									
Notch1	0.8	0.5	0.2	0.4	0.3	0.4	0.1	0.2	1.2
Notch2	0.6	1.1	0.4	0.6	0.6	0.9	0.8	0.9	0.9
Notch3	1.0	**3.0**	**1.5**	1.4	**1.6**	0.0	1.0	1.1	**2.5**
Notch4	Not detectable			Not detectable			Not detectable		
NOTCH LIGANDS									
Jag1	0.5	**3.0**	0.4	0.6	0.9	0.8	0.9	0.7	**4.3**
Jag2	**2.7**	**1.6**	**3.9**	**2.4**	**1.8**	**3.0**	**6.1**	**7.0**	**3.7**
Dll1	0.5	0.9	1.2	0.7	0.2	0.4	0.1	0.1	0.4
Dll3	Not detectable			Not detectable			0.3	0.2	0.2
Dll4	Not detectable			0.5	0.8	0.7	0.8	**5.3**	**4.6**
NOTCH TARGET GENES									
Hey1	**1.7**	**2.6**	**1.7**	0.9	0.9	**2.4**	0.5	0.7	1.0
Hey2	0.5	0.5	0.7	0.2	0.2	0.2	Not detectable		
Hes1	0.5	1.0	1.3	0.7	0.2	0.2	0.2	0.3	1.0
Hes2	Not detectable			**1.8**	**5.2**	1.4	Not detectable		
Hes5	Not detectable			Not detectable			Not detectable		
Hes6	1.4	1.0	0.7	0.8	0.6	0.5	0.3	1.3	1.2

Note: Gene expression was calculated relative to 18S rRNA and normalized by the transcript levels found in cells exposed to 20% O_2_ at the respective time points. Expression values ≥ 1.5-fold have been marked in bold to indicate significant upregulation as compared to normoxic control.

We, therefore, expanded our analysis of Notch response during hypoxia to gliomaspheres. U87MG cells were cultured till 10 days in serum-free tumor sphere medium or normal DMEM under normoxia (20% O_2_), moderate hypoxia (2% O_2_) and severe hypoxia (0.2% O_2_). Sphere medium resulted in gliomasphere formation under all O_2_ concentrations (S3B Fig), with more spheres per well in normoxia than moderate or severe hypoxia (S4 Fig). A possible explanation could be less rate of cell growth/proliferation in low oxygenation than under optimum oxygenation which is likely to reduce sphere growth/formation. Gliomasphere experiment was also tried with U373MG cell line at the same O_2_ concentrations and culture conditions. Although cell aggregation was observed by 5^th^/6^th^ day, it did not develop into fully-formed spheres even by days 9–10 (S5 Fig). The sphere analysis was, therefore, carried out only in U87MG cells.

On day 10, transcript levels of Notch1, Notch2, Notch3, Dll1 and Hey2 were found to be upregulated ≥ 1.5-fold in the gliomaspheres under normoxia (between 1.5–3.5-fold) and even greater under severe or moderate hypoxia (between 1.8–10.1-fold), in comparison to normoxic monolayer cells (Table 8). Hey1 and Hes1 were upregulated in severe and/or moderate hypoxia (up to 2.5 and 4.2-fold, respectively) above the levels found in normoxic spheres. Hes6 showed greater overexpression in the normoxic spheres (2.3-fold) than hypoxic spheres (1.7-fold). By and large, gliomaspheres under normoxia showed upregulation of Notch signaling molecules as compared to monolayers, which increased further in most cases upon exposure to hypoxia. Similarly, we also found increased protein expression of Notch1-cleaved (Notch-intracellular domain (NICD) i.e., the activated receptor that is known to act as a transcription factor [15]) in normoxic gliomaspheres which further enhanced in moderate hypoxia, demonstrating the increasing trend of Notch pathway activation (S3B Fig). Hes1 protein was more upregulated in hypoxic gliomaspheres than hypoxic monolayers. The 10-day Notch response in monolayer cells exposed to severe hypoxia was similar to that found in 3-day severe hypoxia exposure (Tables 7 and 8).

**Table 8:** mRNA fold expression ratios of hypoxia markers and Notch signaling genes in U87MG monolayer and gliomasphere cultures exposed to 20%, 2% and 0.2% O_**2**_ at day 10.

Genes	U87MG (at day 10)					
	Monolayer culture cells			Gliomasphere culture cells		
	20% O_2_ (control)	2% O_2_	0.2% O_2_	20% O_2_	2% O_2_	0.2% O_2_
HYPOXIA MARKERS:						
HIF-1α	1.0	1.4	**1.9**	1.4	1.3	0.5
CA9	1.0	**111.2**	**211.7**	0.5	**72.7**	**237.6**
PGK1	1.0	**2.0**	**6.0**	1.4	**2.1**	**3.7**
NOTCH SIGNALING GENES:						
NOTCH RECEPTORS						
Notch1	1.0	1.1	0.7	**2.1**	**1.7**	**2.5**
Notch2	1.0	0.6	1.1	**1.5**	**1.8**	**3.7**
Notch3	1.0	0.5	**1.7**	**3.5**	**6.2**	**10.1**
Notch4	Not detectable					
NOTCH LIGANDS						
Jag1	1.0	0.7	**2.5**	0.9	0.8	**1.5**
Jag2	1.0	0.8	0.2	0.3	0.4	0.9
Dll1	1.0	0.5	**1.8**	**1.5**	**1.6**	**1.8**
Dll3	Not detectable					
Dll4	Not detectable					
NOTCH TARGET GENES						
Hey1	1.0	0.7	**2.3**	1.1	**1.5**	**2.5**
Hey2	1.0	0.5	**2.8**	**1.5**	**1.9**	1.3
Hes1	1.0	0.5	0.9	0.7	0.7	**4.2**
Hes2	Not detectable					
Hes5	Not detectable					
Hes6	1.0	0.5	1.4	**2.3**	**1.7**	0.8

Note: Gene expression was calculated with respect to at least 2 internal control references (18S rRNA, POLR2A and PPIA) and normalized by the transcript levels found in monolayer cells exposed to 20% O_2_ (day 10). Expression values ≥ 1.5-fold have been marked in bold to indicate significant upregulation as compared to normoxic control.

Unlike primary tumors, where HIF-1α expression correlated significantly with the degree of hypoxia, in U87MG gliomaspheres, HIF-1α did not show correlation with hypoxia (Table 8; S3B Fig). However, the levels of CA9 and PGK1 (target genes of HIF-1α and part of the five-hypoxia marker set identified in primary tumors) showed progressive rise with increasing severity of hypoxia. Also, as a stemness marker, Sox2 protein was assessed. In comparison to normoxic monolayer cells, Sox2 was found to be elevated in normoxic spheres and was further increased by moderate hypoxia (S3B Fig).

Thus, we concluded that U87MG gliomaspheres cultured in moderate/severe hypoxia correlate best with the primary tumors in terms of increased augmentation of common Notch genes i.e., Notch1, Dll1, Hes1, Hes6, Hey1 and Hey2 (Table 8; S3B Fig). These constitute the Notch-axis that is most associated with hypoxia in GBM (S6 Fig). Since tumor spheres mimic the *in-vivo* tumor microenvironment [37] it is probable that the influence of hypoxia on Notch signaling in gliomaspheres is comparable to that in primary GBMs, as demonstrated by our findings.

## Discussion

The published literature on GBM as well as other tumors lacks a holistic view of Notch signaling in relation to hypoxia and its prognostic significance. Moreover, in cultured glioma cells, the relationship of this pathway with moderate (2% O_2_) and severe (0.2% O_2_) hypoxia is unclear. Here, we have evaluated the expression of fifteen Notch genes (Notch1–4, Dll1/3/4, Jag1/2, Hes1/2/5/6 and Hey1/2) and seven hypoxia markers (HIF-1α, PGK1, VEGF, CA9, GLUT1, EPO and OPN) in 35 primary human GBM specimens and determined the molecules defining the hypoxia-Notch signaling axis.

We observed upregulated mRNA of HIF-1α, its transcriptional targets- PGK1, VEGF and CA9; and OPN in the GBM samples studied with respect to normal brain, demonstrating existence of hypoxia in these tumors [44,45]. Presence of hypoxia was further confirmed by increased HIF-1α and VEGF proteins in majority of the tumors analyzed by immunohistochemistry. Among Notch signaling components, Notch1, Notch3, Jag1, Dll1, Hes1, Hes6 and Hey1 were overexpressed at the mRNA level in our dataset.

Even though the intratumoral distribution of hypoxia may be variable within a given sample [46], the specimens that we received, owing to the nature of surgery of gliomas in our institution, were very small and only from a single region of the tumor. This enabled us to assess the correlation between the degree of hypoxia and Notch induction from a limited tissue portion, thereby, providing an insight into the regional associations of signaling molecules which is likely to hold constant. Hence, the regional correlations between hypoxia and Notch pathway thus obtained are likely to remain less variable and would depend on the relative degree of hypoxia in another region of the same tumor. Therefore, for the want of a better parameter we have used these associations as representative of the tumor notwithstanding the intratumoral heterogeneity in GBM. Another evidence that supports the use of small tumor region in our study comes from the statistically significant association obtained between the hypoxia-Notch gene signature and patient survival. It indicates that in spite of the intratumoral heterogeneity, the gene signature is likely to be representative of the tumor.

Prior *in-vitro* studies with monolayer cultures have demonstrated cooperation between hypoxia and Notch signaling in mouse cells through recruitment of HIF-1α to Hey1/Hey2 promoters [19,20,21]. In order to examine the relationship between hypoxia and Notch pathway in GBM tumors, we calculated Spearman’s correlation between the expression of hypoxia markers and Notch molecules. Of all hypoxia markers, HIF-1α, PGK1, OPN and VEGF showed the most significant, positive correlation coefficients with the maximum number of Notch genes. Additionally, as reinforcement to the known function of HIF-1α, we found positive correlation of its expression with PGK1, OPN, VEGF and EPO expression, which is consistent with their direct regulation by HIF-1α [9,10]. PGK1, VEGF and OPN also exhibited high coefficients with the rest of the hypoxia markers implying inter-relatedness.

Although the initial and effective mechanism for increased activity of HIF-1α is by stabilization of the protein, there have been reports of HIF-1α mRNA being increased in cells in response to hypoxia as well as other stresses, eg. tumor inflammation, invasiveness, metastasis, etc. [47,48,49,50,51]. Primary tumors are resident *in-situ* for a long time before surgery. Hence, in order to achieve sustained high activity of HIF-1α, there is the possible requirement for increased mRNA expression. In addition, the high correlation of mRNA expression of HIF-1α with that of known hypoxia markers like PGK1 and VEGF as observed by us in this study also underscores the link between hypoxia, HIF-1α and the hypoxia markers. Also, technically, quantitative real-time PCR is better able to reflect changes in expression levels as opposed to either Western blot followed by densitometry or immunohistochemistry, which are at best semi-quantitative methods.

In gene cluster analysis, HIF-1α, PGK1 and OPN showed the best association with Notch induction since the GBM samples ordered by their individual expression projected greater Notch genes’ upregulation in the high tertile versus low tertile (p ≤ 0.05). The robustness of this semi-supervised strategy was verified by visualization of increased transcripts of HIF-1α target genes in the high HIF-1α tertile, emphasizing HIF-1α function. On carrying out PCA as corroboration to cluster tumors by HIF-1α and PGK1 expression, the weighted summation of selected Notch genes for GBM scores was able to extract homogenous and well-differentiated clusters of high and low tertiles in 2D and 3D plots. Thus, GBM tertiles defined by differential Notch genes’ expression are verifiable as stable GBM sub-groups, depicting the positive correlation between hypoxia and Notch pathway. Further validation of this association was achieved through sensitivity and specificity analysis; and logistic regression models in which individual expression of HIF-1α, PGK1, OPN (and VEGF) was found to be diagnostic for the increased expression of maximum number of Notch genes.

Collectively, all the validation tests employed established the Notch subset comprising of Notch1/Dll1/Hes1/Hes6/Hey1/Hey2 as maximally associated with overexpression of hypoxia markers and therefore, with hypoxia, in GBM tumors. In addition to the single predictors thus obtained (HIF-1α, PGK1, OPN and VEGF), we sought to determine a combination of hypoxia markers that would serve as a predictor of upregulated Notch signaling. Logistic regression analysis identified a five-marker set (HIF-1α/PGK1/VEGF/CA9/OPN) to be associated significantly with the maximum number of Notch genes by the highest correlation coefficient values. Notably, this set associated with almost the same genes as HIF-1α, the foremost single predictor, and also had better predictability for the Notch subset. This study for the first time highlights the potential of a combination of hypoxia markers as a predictor of increased Notch signaling in GBM.

Kaplan-Meier survival plots allow identification of putative clinically relevant genes as new therapeutic targets. We observed significant correlation of poor patient survival with upregulation of components of the observed hypoxia-Notch axis (HIF-1α; p = 0.0205, OPN; p = 0.00172 and Hes1; p = 0.0462) in the available samples. Importantly, the correlation was statistically more significant when the genes were analyzed in combination (HIF-1α/OPN/Hes1; p = 0.00081), reflecting the greater potential of a combinatorial prognostic indicator over individual markers. The association of these combinatorial markers with patient survival also emphasizes the biological relevance of the components of the hypoxia-Notch axis as identified by us. Multi-gene panels have previously been discussed for their improved accuracy and predictive value in prognosis as they may prospectively identify the GBM patients most amenable to conventional therapy and select subgroups that would particularly benefit from additional agents [52,53]. Our hypoxia-Notch subset may be envisioned as a potential predictor for utilization in prognosis similar to other real-time RT-PCR based multi-gene expression assays like DecisionDX-GBM [52]. However, a larger sample size is required to validate its predictive power.

To further understand the hypoxia-Notch relationship, we studied the expression profile of Notch molecules in GBM cell line monolayers cultured under low oxygen tensions. As compared to primary tumors, analysis in U87MG/A172/U373MG monolayer cells revealed a limited Notch response confined to fewer Notch genes and lesser extent of upregulation, even though the hypoxia markers were induced several-fold under severe hypoxia at 24/48/72 hours. The differences observed in the GBM monolayer cell cultures may be attributed to the adaptive genetic alterations found in established cell lines resulting from years of passage and selection pressure of *in-vitro* culture conditions [54]. Moreover, Notch signaling is known to differ with the cell type [14,16]. More recently, tumor spheroids have been discussed as models of cancer cells enriched in stemness properties and have disclosed differences in cellular behaviour of the two types of culture systems [54,55]. Owing to the extensive variability in Notch response in the hypoxic monolayer cultures as compared to resected GBM tissues, we examined the expression of Notch components in U87MG gliomaspheres cultured in moderate/severe hypoxia. Hypoxic environment in the spheres was confirmed by progressive rise of CA9 and PGK1 from moderate to severe hypoxia, although HIF-1α protein displayed an anomalous pattern [41,42,43] similar to that observed in monolayer cells. Presence of stem-like cells was reflected by increased Sox2 in the normoxic spheres. Hypoxia was seen to upgrade this stemness property in both gliomaspheres and monolayer cells. Gliomaspheres displayed upregulation of Notch1, Notch2, Notch3, Dll1, Hey2 and Hes6 under normoxia as compared to normoxic monolayers, which is possible since gliomaspheres are intrinsically different from monolayers with regard to stemness properties and cell signaling [55]. Also, gliomaspheres are often composed of approximately >100 cells due to which there exists an oxygen diffusion gradient that could result in hypoxic centres [7]. However, overall, it is apparent that hypoxic gliomaspheres mimic the *in-vivo* tumor condition better than the other tissue culture model as the upregulation of Notch genes (Notch1, Notch2, Notch3, Dll1, Hes1, Hey1 and Hey2) is further enhanced upon exposure to hypoxia. The increased expression of Notch1-cleaved and downstream molecules (Hes1, Hey1 and Hey2) in hypoxic spheres is an indication of Notch pathway activation. Hence, similar components of the hypoxia-activated Notch response in primary GBM specimens and gliomaspheres are suggestive of the greater similarity of tumors with spheres than monolayer cultures. Gliomaspheres could not be established using U373MG cell line. A recent study has also reported the inability of U373MG spheres to persist in culture [56].

Thus, the Notch pathway molecules consistently activated in response to hypoxia in the human GBM tumors were delineated in gliomaspheres. While our comprehensive tumor analysis has identified the key components of hypoxia-Notch axis in glioma patients, the hypoxia-exposed gliomaspheres would provide a close *in-vitro* model for future studies on mechanisms of activation and points of intervention.

As the current focus in medical arena is therapy determined by molecular sub-grouping, an understanding of the molecular relationship governing Notch and hypoxia in GBM is clinically relevant. Our findings establish a signaling axis whose upregulation represents a potential marker of Notch signaling activation under hypoxia in GBM. The hypoxia-Notch gene subset (HIF-1α/PGK1/VEGF/CA9/OPN-Notch1/Dll1/Hes1/Hes6/Hey1/Hey2) identified by us might hold prognostic implication and offer new opportunities in tumor sub-classification and targeted therapy.

## Supporting Information

S1 Fig Immunohistochemical analysis of hypoxia markers in paraffin-embedded GBM tumors. Representative photomicrographs (at 20x magnification) of immunohistochemical analysis depicting positive staining for HIF-1α and VEGF in GBM samples have been shown. Prominent nuclear staining of HIF-1α was observed in the tumor cells of all GBM samples (100%) analyzed, especially in perinecrotic areas (indicated by black arrow), representing HIF-1α protein stabilization in hypoxic areas. HIF-1α-regulated marker, VEGF, was primarily distributed in the cytoplasm of vascular and tumor cells in 14/16 GBM cases (88%). Both HIF-1α and VEGF displayed upregulation in GBM tissue as compared to the negatively stained normal brain samples. The table shows results of HIF-1α and VEGF staining in 17 paraffin-embedded GBM samples and normal brain. + refers to positive immunostaining;—refers to negative immunostaining; N.A.: data not available due to lack of tissue sample or presence of necrotic tissue.
(TIF)

S2 Fig Heat maps showing cluster analysis of Notch genes in GBM tumor tertiles based on VEGF and CA9 expression. Clustering of Notch pathway genes in GBMs sorted in decreasing order of (A) VEGF or (B) CA9 expression. Gene expression found significantly different across the high and low tertiles (p ≤ 0.05) has been indicated by an asterisk (*). Only 1/15 Notch genes displayed greater upregulation in the high VEGF/CA9 GBM tertile.
(TIF)

S3 Fig Expression of hypoxia markers and Notch signaling molecules in monolayer GBM cell lines and gliomasphere cultures exposed to hypoxia. **(A)** Western blot analysis of hypoxia markers (CA9 and HIF-1α) and Notch signaling molecules (Notch3, Hey1 and Hes1) in U87MG monolayer culture cells and HIF-1α, Notch3 and Hes1 in U373MG monolayer culture cells at 24, 48 and 72 hours (h) of exposure to severe hypoxia (0.2% O_2_) as compared to normoxia (N; 20% O_2_). β-actin was used as the loading control. Hypoxia-induced increase in expression of CA9; and HIF-1α (in U373MG but not in U87MG) was observed in the GBM cell line monolayer cultures. However, selected Notch genes (Notch3, Hes1 and Hey1) were upregulated in response to severe hypoxia in U87MG/U373MG. **(B)** Photomicrographs of U87MG cells exposed to 20%, 2% and 0.2% O_2_ on day 10 (at 10x magnification). The left panel represents cells cultured as monolayers while the right panel represents cells cultured as gliomaspheres. Arrows point to the spheres formed in tumor sphere medium at all O_2_ concentrations. The figure below shows the Western blot analysis of hypoxia markers (PGK1 and HIF-1α), stemness marker (Sox2) and Notch signaling molecules (Notch1-cleaved and Hes1) in U87MG cells at day 10 upon culture in normal (N) and tumor sphere (S) media and exposure to normoxia (20% O_2_), moderate hypoxia (2% O_2_) or severe hypoxia (0.2% O_2_). β-actin was used as the loading control. Expression of PGK1, but not HIF-1α, was found to increase with the increase in severity of hypoxia in both monolayer cells and gliomaspheres. Sox2 was more upregulated in normoxic as well as hypoxic gliomaspheres than normoxic monolayer cells. The expression of Notch signaling read-outs viz. Notch1-cleaved and Hes1 was found to be more enhanced in hypoxic spheres than normoxic monolayer cells.
(ZIP)

S4 Fig *In-vitro* gliomasphere culture using U87MG cell line and exposure to hypoxia. Photomicrographs of U87MG cells exposed to 20%, 2% and 0.2% O_2_ on days 6, 7, 8, 9 and 10 (at 4x magnification). The left panel represents cells cultured as monolayers while the right panel represents cells cultured as gliomaspheres. Arrows point to sphere formation which began by the 6^th^ day at all O_2_ concentrations in tumor sphere medium. As shown in the table, the average number of spheres formed per well on day 10 was noted to be more in normoxia than moderate or severe hypoxia.
(TIF)

S5 Fig *In-vitro* gliomasphere culture using U373MG cell line and exposure to hypoxia. Photomicrographs of U373MG cells exposed to 20%, 2% and 0.2% O_2_ on days 1, 6 and 9 (at 10x magnification). The left panel represents cells cultured in normal DMEM while the right panel represents cells cultured in tumor sphere medium. Arrows point to cell aggregation which began by the 6^th^ day at all O_2_ concentrations in tumor sphere medium. This aggregation, however, did not persist by day 9.
(TIF)

S6 Fig Possible Notch signaling axes operating in hypoxia in GBM tumors and U87MG gliomaspheres. Similarities in Notch pathway augmentation in response to hypoxia were observed in terms of upregulation of Dll1, Notch1, Hes1, Hes6, Hey1 and Hey2 in the studied tumor samples and gliomaspheres.
(TIF)

S1 Table Details of primers for hypoxia markers, Notch receptors, Notch ligands and Notch target genes used for real-time PCR. (DOC)

S2 Table Details of primers for internal control references used for real-time PCR. (DOC)

S3 Table Fold expression ratios of hypoxia markers and Notch signaling genes in 35 GBM samples. (DOC)

S4 Table Expression values of hypoxia markers in 35 GBM samples grouped by tertiles in decreasing order of HIF-1α expression. (DOC)

S5 Table Expression values of Notch signaling genes in 35 GBM samples grouped by tertiles in decreasing order of HIF-1α expression. (DOC)

S6 Table Expression values of Notch signaling genes in 35 GBM samples grouped by tertiles in decreasing order of PGK1 expression. (DOC)

S7 Table Expression values of Notch signaling genes in 35 GBM samples grouped by tertiles in decreasing order of OPN expression. (DOC)

S8 Table Expression values of Notch signaling genes in 35 GBM samples grouped by tertiles in decreasing order of VEGF expression. (DOC)

S9 Table Expression values of Notch signaling genes in 35 GBM samples grouped by tertiles in decreasing order of CA9 expression. (DOC)

S10 Table Summary of sensitivity and specificity of CA9 and VEGF expression in diagnosis of Notch genes’ overexpression in 35 GBM tumors. (DOC)

S11 Table Summary of results of linear regression analysis using all possible combinations of hypoxia markers (parts (i)-(xiii)) as independent variables for their combined association with the expression level of individual Notch genes. (DOC)

S12 Table Survival time of 21 GBM patients with the corresponding mRNA expression of the identified components of hypoxia-Notch signaling axis. (DOC)

S1 File Supplementary data text. Supplementary Materials and Methods.
(DOC)

S2 File R analysis supplemental content. . zip folder containing the R script master code and input data files for performing PCA and Kaplan-Meier survival analysis.
(ZIP)
