# Supplementary figures and images for: A Combined Gene Signature of Hypoxia and Notch Pathway in Human Glioblastoma and Its Prognostic Relevance
Source: PLoS One. 2015 Mar 3;10(3):e0118201. doi: 10.1371/journal.pone.0118201 (PMC4348203; doi:10.1371/journal.pone.0118201)

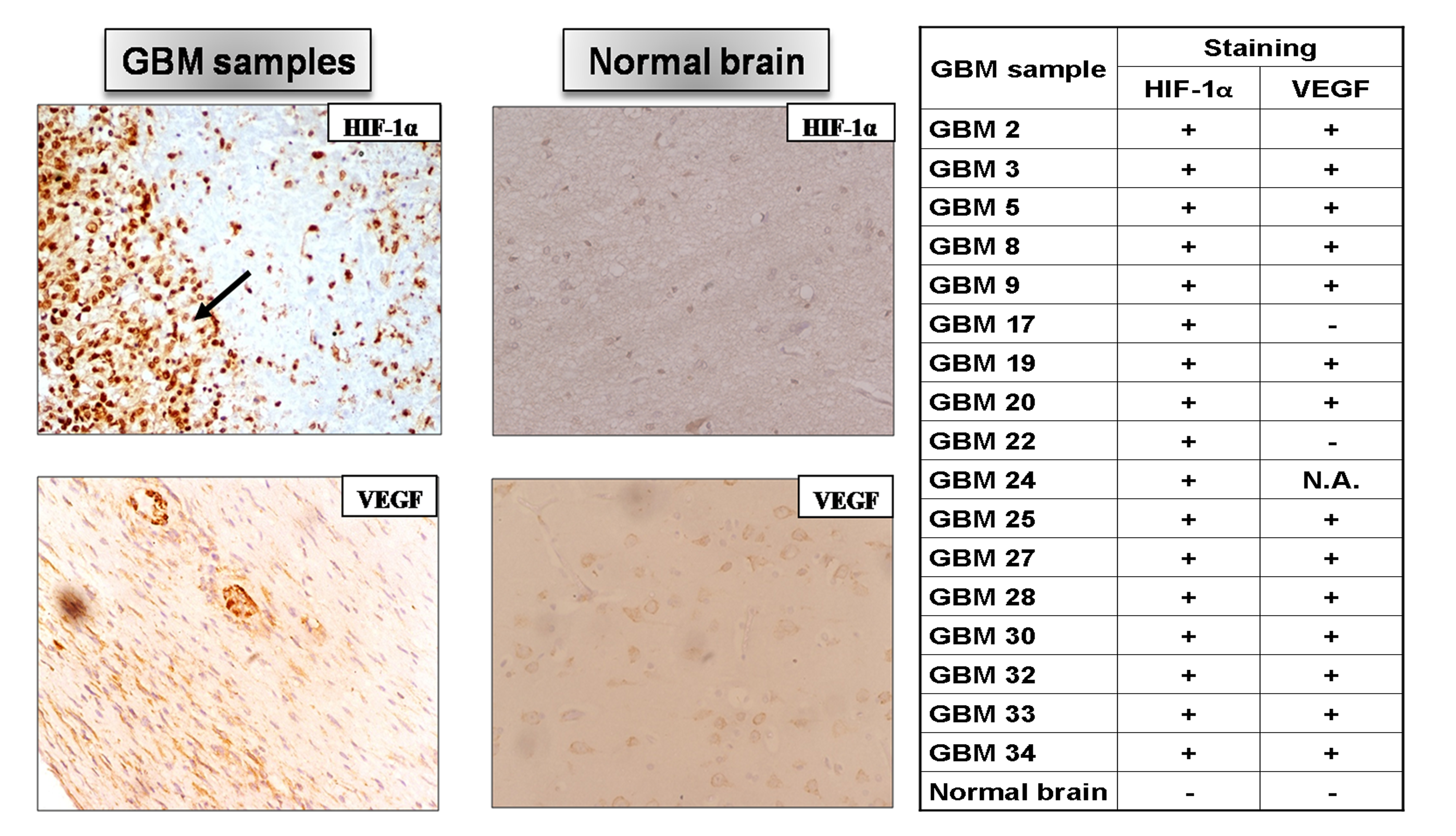

Supplement: S1 Fig — Representative photomicrographs (at 20x magnification) of immunohistochemical analysis depicting positive staining for HIF-1α and VEGF in GBM samples have been shown. Prominent nuclear staining of HIF-1α was observed in the tumor cells of all GBM samples (100%) analyzed, especially in perinecrotic areas (indicated by black arrow), representing HIF-1α protein stabilization in hypoxic areas. HIF-1α-regulated marker, VEGF, was primarily distributed in the cytoplasm of vascular and tumor cells in 14/16 GBM cases (88%). Both HIF-1α and VEGF displayed upregulation in GBM tissue as compared to the negatively stained normal brain samples. The table shows results of HIF-1α and VEGF staining in 17 paraffin-embedded GBM samples and normal brain. + refers to positive immunostaining;—refers to negative immunostaining; N.A.: data not available due to lack of tissue sample or presence of necrotic tissue. (TIF) [file pone.0118201.s001.tif]

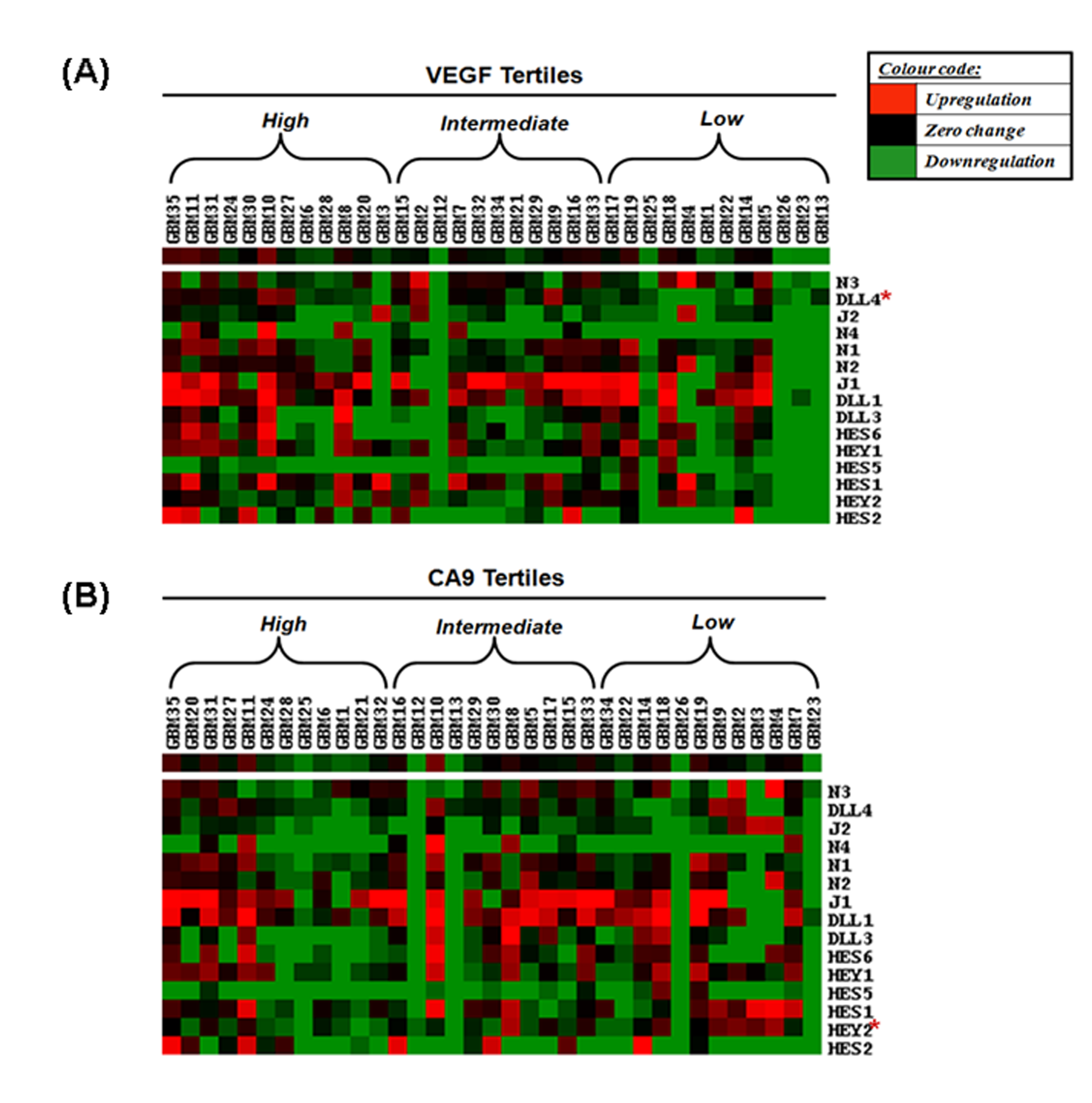

Supplement: S2 Fig — Clustering of Notch pathway genes in GBMs sorted in decreasing order of (A) VEGF or (B) CA9 expression. Gene expression found significantly different across the high and low tertiles (p ≤ 0.05) has been indicated by an asterisk (*). Only 1/15 Notch genes displayed greater upregulation in the high VEGF/CA9 GBM tertile. (TIF) [file pone.0118201.s002.tif]

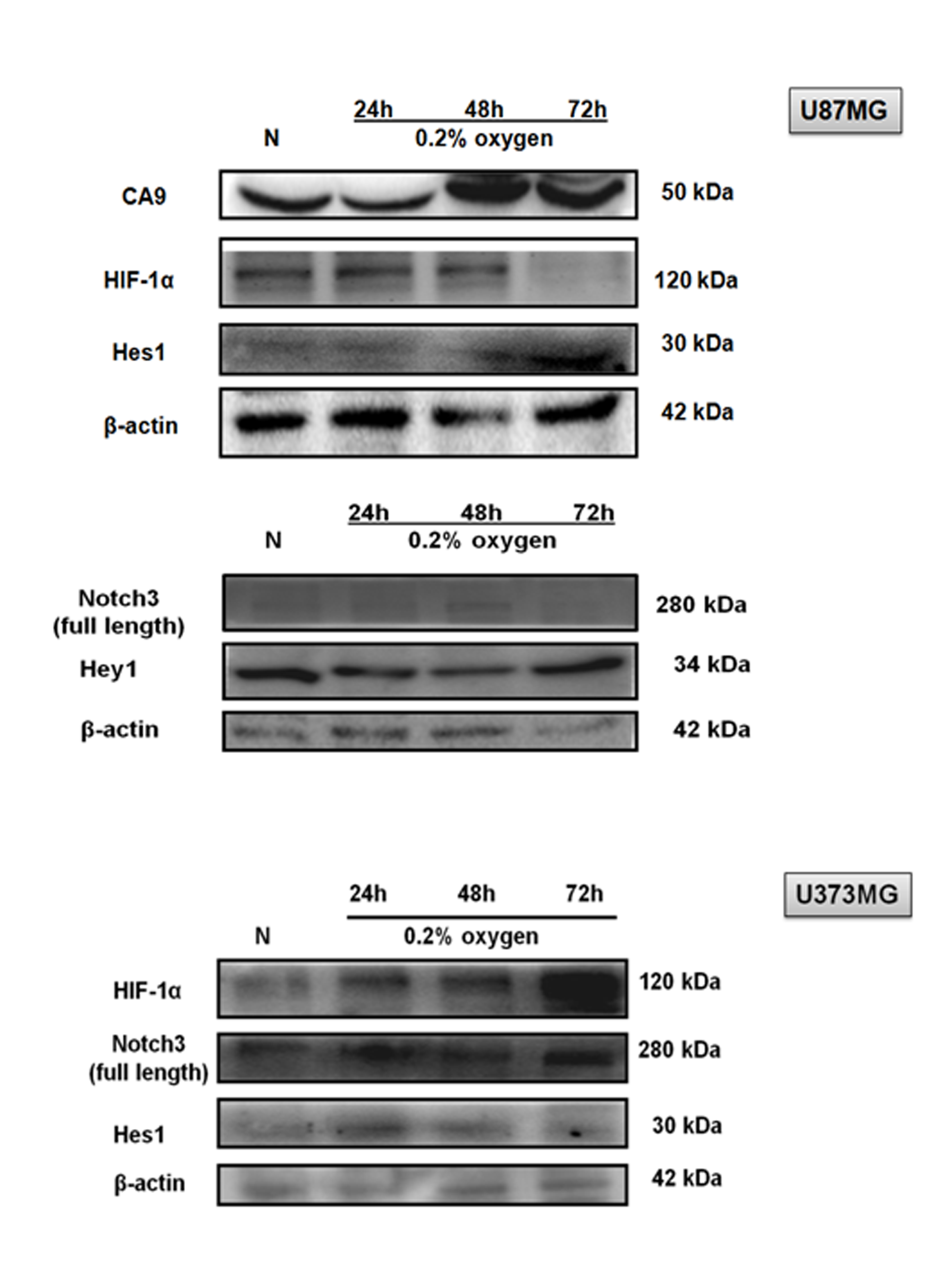

Supplement: S3 Fig — (A) Western blot analysis of hypoxia markers (CA9 and HIF-1α) and Notch signaling molecules (Notch3, Hey1 and Hes1) in U87MG monolayer culture cells and HIF-1α, Notch3 and Hes1 in U373MG monolayer culture cells at 24, 48 and 72 hours (h) of exposure to severe hypoxia (0.2% O2) as compared to normoxia (N; 20% O2). β-actin was used as the loading control. Hypoxia-induced increase in expression of CA9; and HIF-1α (in U373MG but not in U87MG) was observed in the GBM cell line monolayer cultures. However, selected Notch genes (Notch3, Hes1 and Hey1) were upregulated in response to severe hypoxia in U87MG/U373MG. (B) Photomicrographs of U87MG cells exposed to 20%, 2% and 0.2% O2 on day 10 (at 10x magnification). The left panel represents cells cultured as monolayers while the right panel represents cells cultured as gliomaspheres. Arrows point to the spheres formed in tumor sphere medium at all O2 concentrations. The figure below shows the Western blot analysis of hypoxia markers (PGK1 and HIF-1α), stemness marker (Sox2) and Notch signaling molecules (Notch1-cleaved and Hes1) in U87MG cells at day 10 upon culture in normal (N) and tumor sphere (S) media and exposure to normoxia (20% O2), moderate hypoxia (2% O2) or severe hypoxia (0.2% O2). β-actin was used as the loading control. Expression of PGK1, but not HIF-1α, was found to increase with the increase in severity of hypoxia in both monolayer cells and gliomaspheres. Sox2 was more upregulated in normoxic as well as hypoxic gliomaspheres than normoxic monolayer cells. The expression of Notch signaling read-outs viz. Notch1-cleaved and Hes1 was found to be more enhanced in hypoxic spheres than normoxic monolayer cells. (ZIP) [file pone.0118201.s003.zip › FIGURE S3(A).tif]

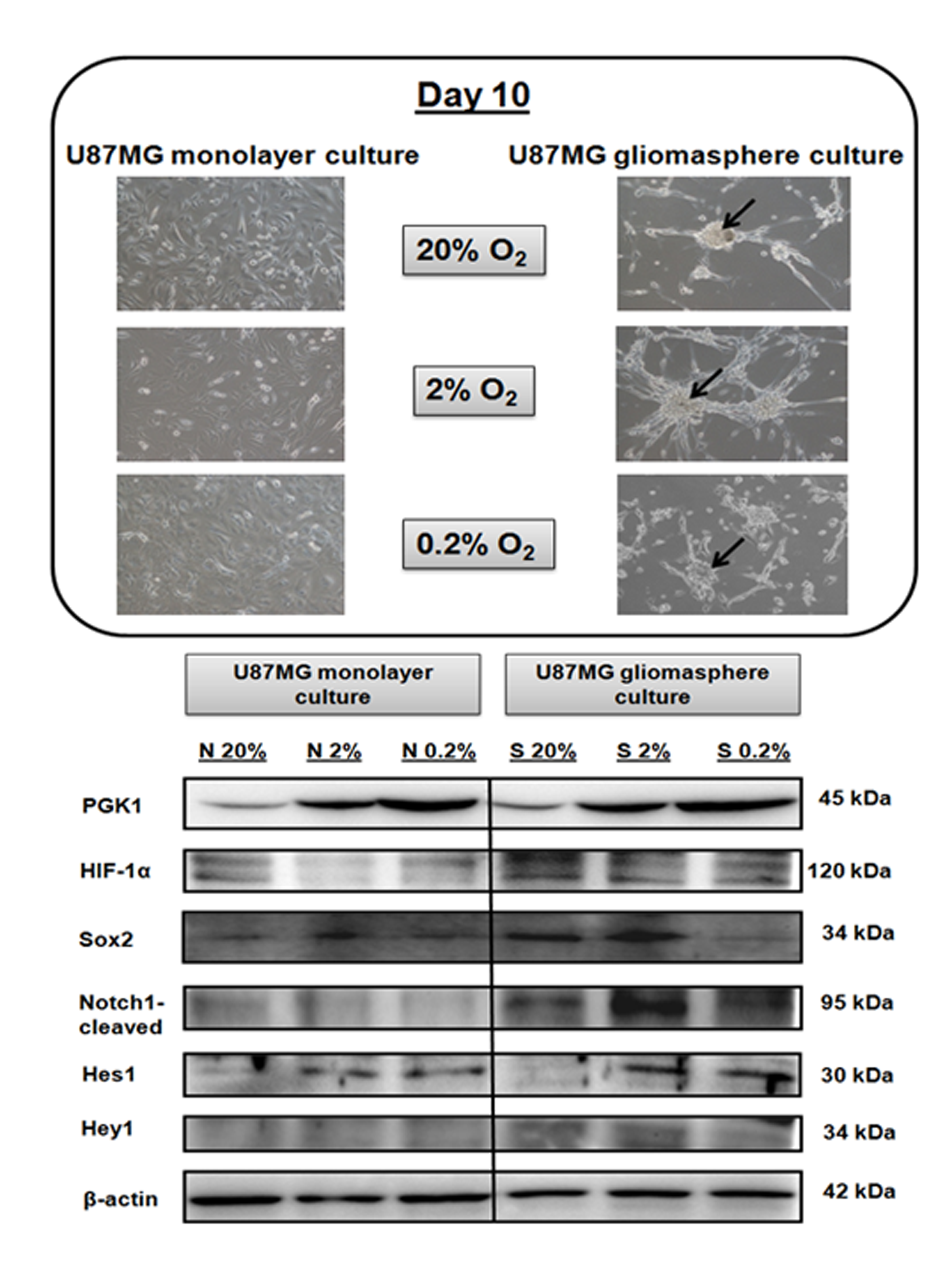

Supplement: S3 Fig — (A) Western blot analysis of hypoxia markers (CA9 and HIF-1α) and Notch signaling molecules (Notch3, Hey1 and Hes1) in U87MG monolayer culture cells and HIF-1α, Notch3 and Hes1 in U373MG monolayer culture cells at 24, 48 and 72 hours (h) of exposure to severe hypoxia (0.2% O2) as compared to normoxia (N; 20% O2). β-actin was used as the loading control. Hypoxia-induced increase in expression of CA9; and HIF-1α (in U373MG but not in U87MG) was observed in the GBM cell line monolayer cultures. However, selected Notch genes (Notch3, Hes1 and Hey1) were upregulated in response to severe hypoxia in U87MG/U373MG. (B) Photomicrographs of U87MG cells exposed to 20%, 2% and 0.2% O2 on day 10 (at 10x magnification). The left panel represents cells cultured as monolayers while the right panel represents cells cultured as gliomaspheres. Arrows point to the spheres formed in tumor sphere medium at all O2 concentrations. The figure below shows the Western blot analysis of hypoxia markers (PGK1 and HIF-1α), stemness marker (Sox2) and Notch signaling molecules (Notch1-cleaved and Hes1) in U87MG cells at day 10 upon culture in normal (N) and tumor sphere (S) media and exposure to normoxia (20% O2), moderate hypoxia (2% O2) or severe hypoxia (0.2% O2). β-actin was used as the loading control. Expression of PGK1, but not HIF-1α, was found to increase with the increase in severity of hypoxia in both monolayer cells and gliomaspheres. Sox2 was more upregulated in normoxic as well as hypoxic gliomaspheres than normoxic monolayer cells. The expression of Notch signaling read-outs viz. Notch1-cleaved and Hes1 was found to be more enhanced in hypoxic spheres than normoxic monolayer cells. (ZIP) [file pone.0118201.s003.zip › FIGURE S3(B).tif]

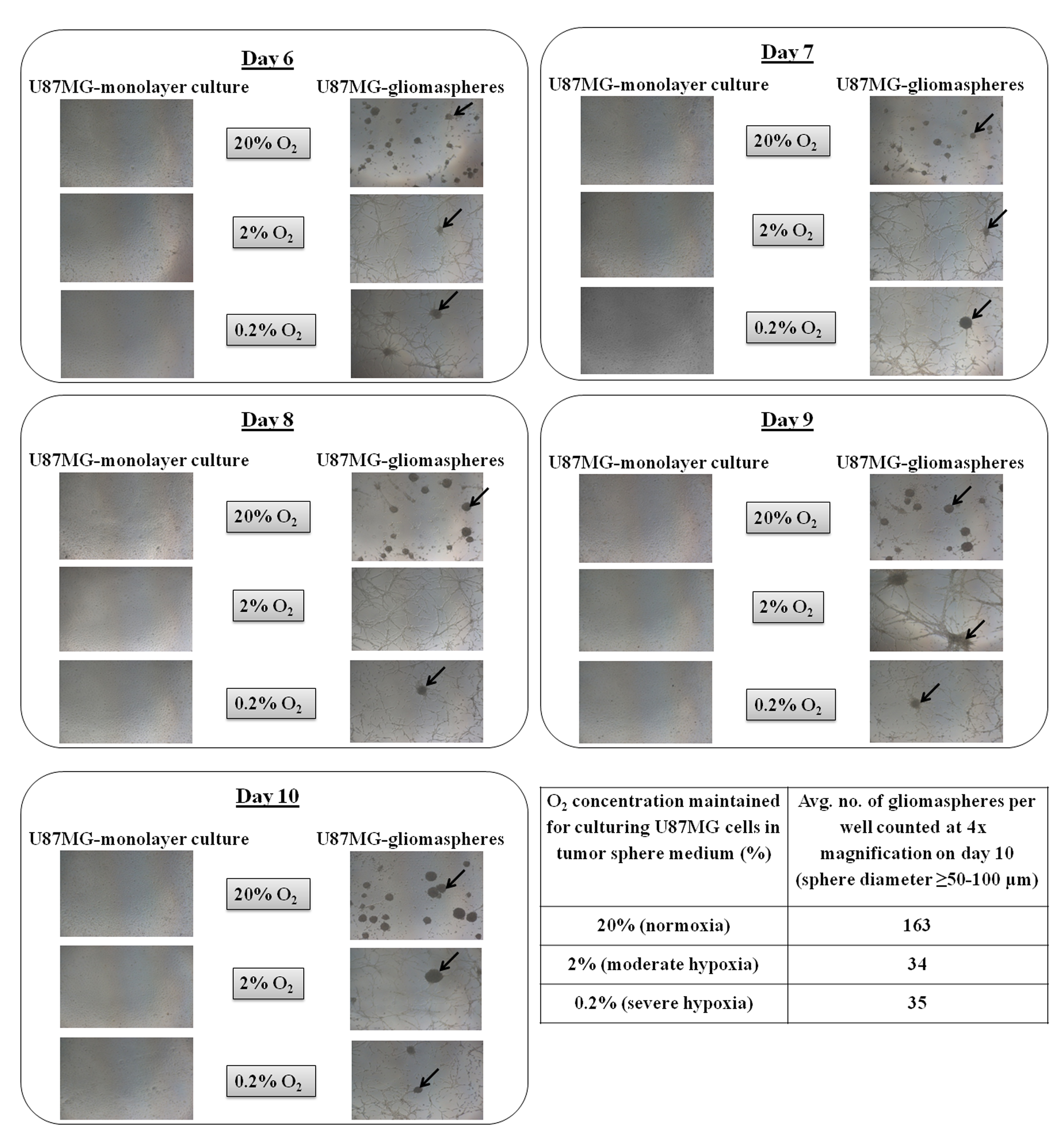

Supplement: S4 Fig — Photomicrographs of U87MG cells exposed to 20%, 2% and 0.2% O2 on days 6, 7, 8, 9 and 10 (at 4x magnification). The left panel represents cells cultured as monolayers while the right panel represents cells cultured as gliomaspheres. Arrows point to sphere formation which began by the 6th day at all O2 concentrations in tumor sphere medium. As shown in the table, the average number of spheres formed per well on day 10 was noted to be more in normoxia than moderate or severe hypoxia. (TIF) [file pone.0118201.s004.tif]

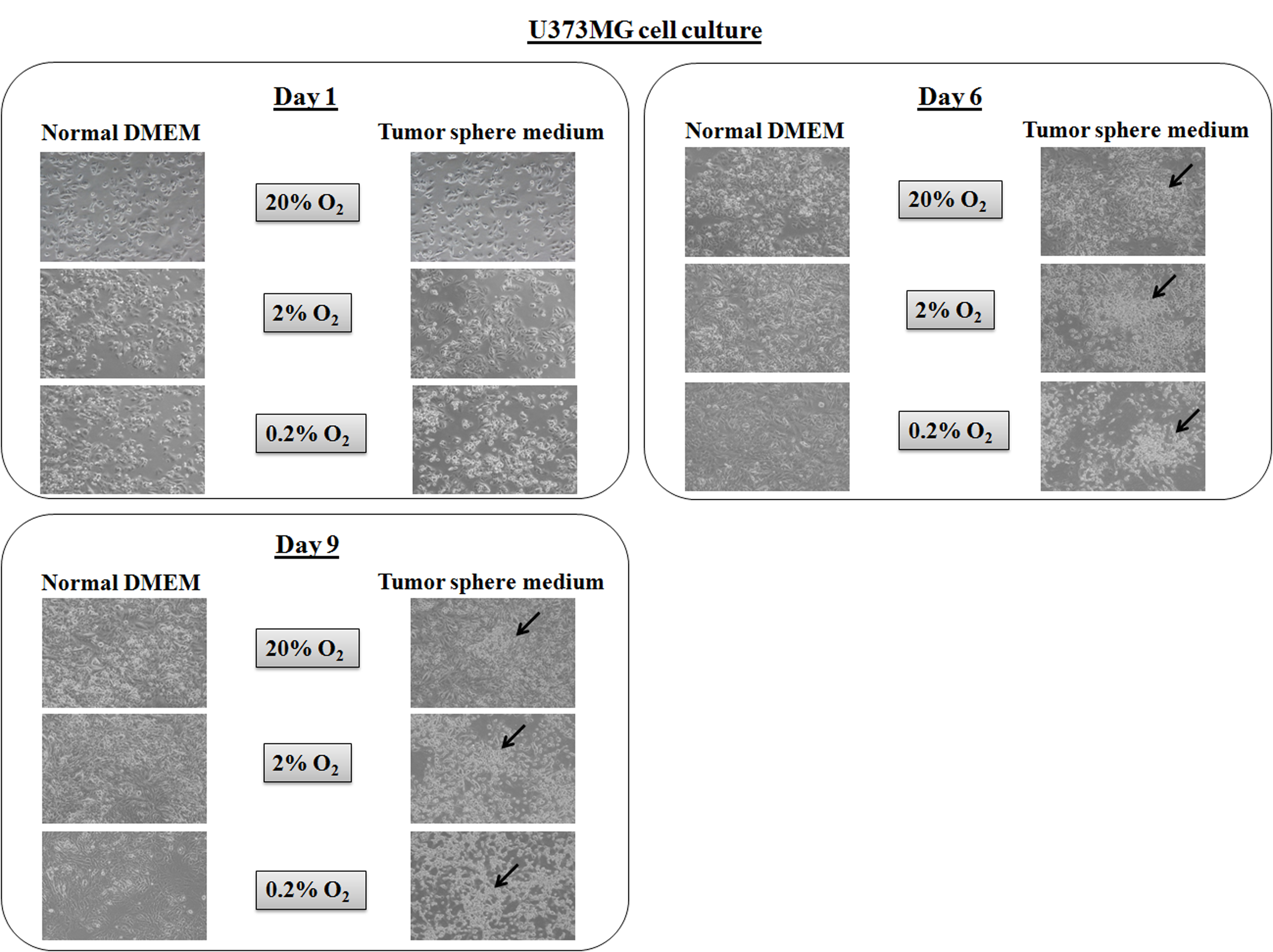

Supplement: S5 Fig — Photomicrographs of U373MG cells exposed to 20%, 2% and 0.2% O2 on days 1, 6 and 9 (at 10x magnification). The left panel represents cells cultured in normal DMEM while the right panel represents cells cultured in tumor sphere medium. Arrows point to cell aggregation which began by the 6th day at all O2 concentrations in tumor sphere medium. This aggregation, however, did not persist by day 9. (TIF) [file pone.0118201.s005.tif]

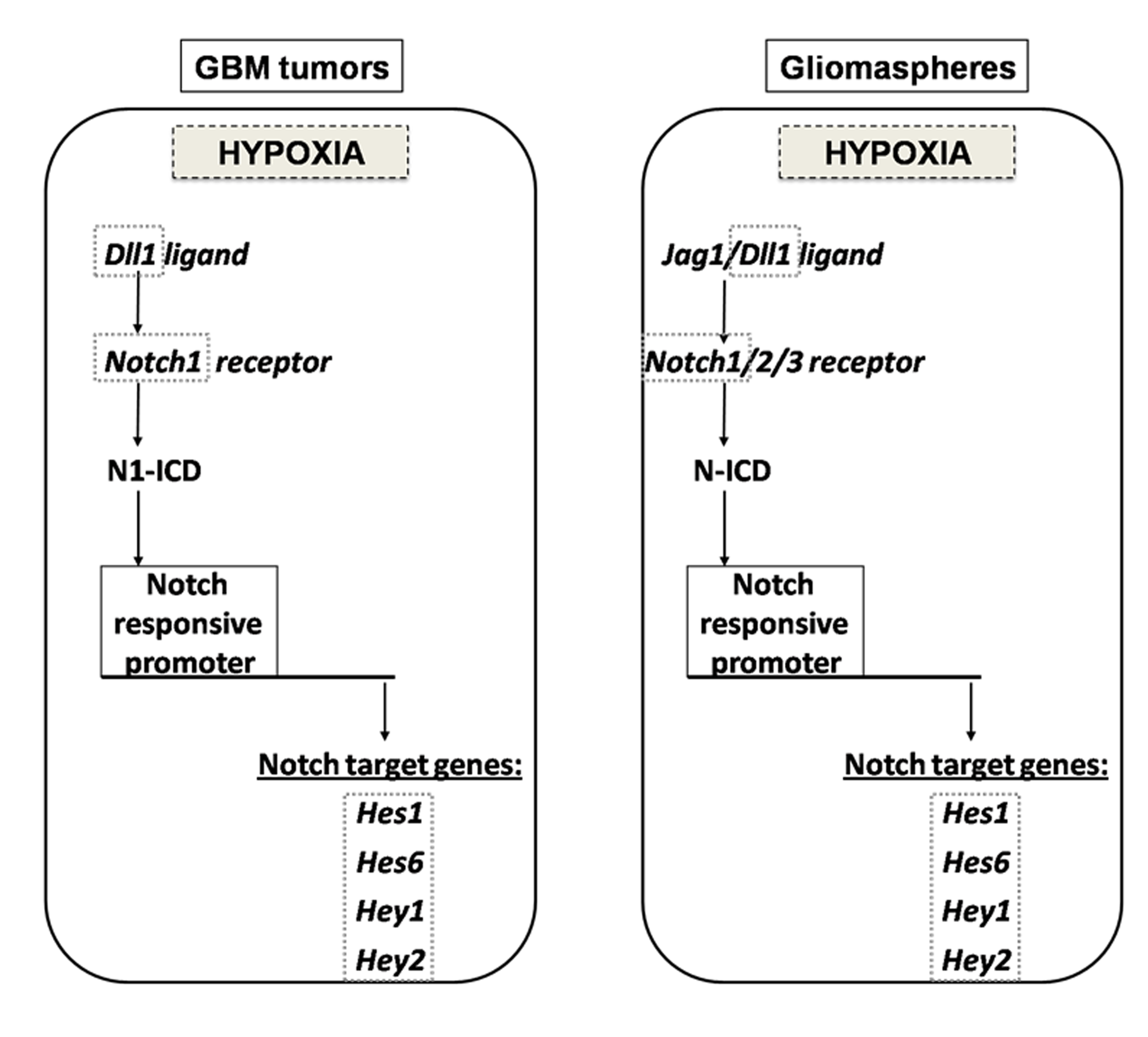

Supplement: S6 Fig — Similarities in Notch pathway augmentation in response to hypoxia were observed in terms of upregulation of Dll1, Notch1, Hes1, Hes6, Hey1 and Hey2 in the studied tumor samples and gliomaspheres. (TIF) [file pone.0118201.s006.tif]
