# Supplementary material for: A Combined Gene Signature of Hypoxia and Notch Pathway in Human Glioblastoma and Its Prognostic Relevance
Source: PLoS One. 2015 Mar 3;10(3):e0118201. doi: 10.1371/journal.pone.0118201 (PMC4348203; doi:10.1371/journal.pone.0118201)
Supplement: S1 Table — (DOC) [file pone.0118201.s007.doc]

**Table S1.** Details of primers for hypoxia markers, Notch receptors, Notch ligands and Notch target genes used for real-time PCR

| ***HYPOXIA MARKERS:*** | | | | | | | | | | | | |
| --- | --- | --- | --- | --- | --- | --- | --- | --- | --- | --- | --- | --- |
| ***Gene*** | ***Primer sequence(5’-3’)*** | | | ***Annealing temperature (^0^C)*** | | ***Product T_m_(^0^C)*** | | ***Primer dimer T_m_(^0^C)*** | | ***Acquisition temperature (^0^C)*** | | ***Amplicon size***  ***(bp)*** |
| **HIF-1α**  For primer | GGGTATAAGAAACCACCTATGACCT | | | 60 | | 83 | | 76 | | 72 or 79 | | 165 |
| Rev primer | atatcccatcaattcggtaattCTT | | |  |  |  |  |  |  |  |  |  |
| **PGK1**  For primer | CCGAGCCAGCCAAAATAGA | | | 59 | | 88 | | 54 | | 72 or 84 | | 243 |
| Rev primer | GCTGGATCTTGTCTGCAACTTTAG | | |  |  |  |  |  |  |  |  |  |
| **VEGF**  For primer | ACCATGAACTTTCTGCTGTCTTG | | | 60 | | 88 | | 56 | | 72 or 84 | | 229 |
| Rev primer | ATGGCTTGAAGATGTACTCGATCTC | | |  |  |  |  |  |  |  |  |  |
| **CA9**  For primer | ctttgaatgggcgagtgatt | | | 62 | | 88 | | 55 | | 72 or 81 | | 184 |
| Rev primer | CTTctgtgctgccttctcatct | | |  |  |  |  |  |  |  |  |  |
| **OPN**  For primer | ACCTGACATCCAGtaccctgat | | | 60 | | 88 | | 76 | | 72 or 84 | | 217 |
| Rev primer | ccgcttatataatctggactgctt | | |  |  |  |  |  |  |  |  |  |
| **EPO**  For primer  Rev primer | GAATGAGAATATCACTGTCCCAGAC | | | 64 | | 91 | | 53 | | 72 or 87 | | 197 |
|  | CACTGACGGCTTTATCCACAT | | |  |  |  |  |  |  |  |  |  |
| **GLUT1**  For primer  Rev primer | CTTCTATTACTCCACGAGCATCTTC | | | 64 | | 92 | | 53 | | 72 or 87 | | 231 |
|  | AGATAGGACATCCAGGGTAGCTG | | |  |  |  |  |  |  |  |  |  |
| ***NOTCH RECEPTORS:*** | | | | | | | | | | | | |
| **Notch1**  For primer | | Gcactttctgtgaggaggacat | 62 | | 91 | | 54 | | 72 or 87 | | 162 | |
| Rev primer | | AGCAGGAGctctctgtgcagt |  |  |  |  |  |  |  |  |  |  |
| **Notch2**  For primer | | GAACACTGGGTCGATGATGAAG | 64 | | 88 | | 78 | | 72 or 84 | | 198 | |
| Rev primer | | CACATCTAACACATCCACCTCCT |  |  |  |  |  |  |  |  |  |  |
| **Notch3**  For primer | | GGGTTCCGCTGTGTGTGT | 64 | | 93 | | 80 | | 72 or 87 | | 177 | |
| Rev primer | | GGAGAGGAGTTCACACTGACG |  |  |  |  |  |  |  |  |  |  |
| **Notch4**  For primer | | ccagagATCCTCATGTGTGTGT | 58 | | 92 | | 77 | | 72 or 88 | | 159 | |
| Rev primer | | gtgggtcCTGTGTAGCCTGTAG |  |  |  |  |  |  |  |  |  |  |
| ***NOTCH LIGANDS:*** | | | | | | | | | | | | |
| **Dll1**  For primer | | ATGTGATGAGCAGCATGGATT | 64 | | 90 | | 54 | | 72 or 87 | | 197 | |
| Rev primer | | GGTGTGTGCAGTAGTTCAGGTC |  |  |  |  |  |  |  |  |  |  |
| **Dll3**  For primer | | TGAGCATGGCTTCTGTGAAC | 62 | | 92 | | 79 | | 72 or 87 | | 211 | |
| Rev primer | | AAAGGACCTGGGTGTCTCACTAC |  |  |  |  |  |  |  |  |  |  |
| **Dll4**  For primer | | GTGACCAAGATCTCAACTACTGC | 62 | | 89 | | 79 | | 72 or 84 | | 163 | |
| Rev primer | | CAGGGGTTGCTGTCACACTC |  |  |  |  |  |  |  |  |  |  |
| **Jag1**  For primer | | CTGCTCAAAGgtctggtgtg | 62 | | 91 | | 52 | | 72 or 87 | | 152 | |
| Rev primer | | ggagactggaagaccgacac |  |  |  |  |  |  |  |  |  |  |
| **Jag2**  For primer | | aggttctgcgatgagtgtgtc | 62 | | 92 | | 81 | | 72 or 87 | | 237 | |
| Rev primer | | gtgctcagCCTTCTCACAGTT |  |  |  |  |  |  |  |  |  |  |
| ***NOTCH TARGET GENES:*** | | | | | | | | | | | | |
| **Hes1**  For primer | | ggatgctctgaagaaagatAGCTC | 60 | | 89 | | 77 | | 72 or 84 | | 145 | |
| Rev primer | | gtacttccccagcacacttgg |  |  |  |  |  |  |  |  |  |  |
| **Hes2**  For primer | | GGCCGGGAGAACTCCAAC | 65 | | 91 | | 54 | | 72 or 87 | | 150 | |
| Rev primer | | CACACAGGCGCTGTAGCC |  |  |  |  |  |  |  |  |  |  |
| **Hes5**  For primer | | CTACCTGAAGCACAGCAAAGC | 65 | | 92 | | 58 | | 72 or 87 | | 157 | |
| Rev primer | | GAAGTGGTACAGCAGCTTCATCT |  |  |  |  |  |  |  |  |  |  |
| **Hes6**  For primer | | AGCTGGAGAACGCCGAAGT | 62 | | 95 | | 81 | | 72 or 87 | | 220 | |
| Rev primer | | atggactcgagcagatggtt |  |  |  |  |  |  |  |  |  |  |
| **Hey1**  For primer | | gccgagatcctgcagatga | 59 | | 87 | | 53 | | 72 or 81 | | 223 | |
| Rev primer | | GCTGGGAAGCGTAGTTGTTG |  |  |  |  |  |  |  |  |  |  |
| **Hey2**  For primer | | AGgctactttgacgcacacg | 59 | | 88 | | 51 | | 72 or 81 | | 153 | |
| Rev primer | | CAAGTGCTGAGATGAGACACAAG |  |  |  |  |  |  |  |  |  |  |
