# Supplementary material for: A Combined Gene Signature of Hypoxia and Notch Pathway in Human Glioblastoma and Its Prognostic Relevance
Source: PLoS One. 2015 Mar 3;10(3):e0118201. doi: 10.1371/journal.pone.0118201 (PMC4348203; doi:10.1371/journal.pone.0118201)
Supplement: S2 Table — (DOC) [file pone.0118201.s008.doc]

**Table S2.** Details of primers for internal control references used for real-time PCR

| ***Reference*** | ***Primer sequence (5’-3’)*** | ***Amplicon size (bp)*** | ***Annealing***  ***temperature (°C)*** |
| --- | --- | --- | --- |
| **18S rRNA:**  For primer  Rev primer | GTAACCCGTTGAACCCCATT | 151 | 58-65 |
|  | CCATCCAATCGGTAGTAGCG |  |  |
| **POLR2A:**  For primer  Rev primer | CATCAAGAGAGTCCAGTTCGG | 96 |  |
|  | CCCTCAGTCGTCTCTGGGTA |  |  |
| **PPIA:**  For primer  Rev primer | CACCGTGTTCTTCGACATTG | 91 |  |
|  | TTCTGCTGTCTTTGGGACCT |  |  |
