# Supplementary material for: A Combined Gene Signature of Hypoxia and Notch Pathway in Human Glioblastoma and Its Prognostic Relevance
Source: PLoS One. 2015 Mar 3;10(3):e0118201. doi: 10.1371/journal.pone.0118201 (PMC4348203; doi:10.1371/journal.pone.0118201)
Supplement: S3 Table — (DOC) [file pone.0118201.s009.doc]

**Table S3.** Fold expression ratios of hypoxia markers and Notch signaling genes in 35 GBM samples

| Sample ID | HIF-1α | PGK1 | VEGF | GLUT1 | OPN | EPO | CA9 | Notch1 | Notch2 | Notch3 | Notch4 | Dll1 | Dll3 | Dll4 | Jag1 | Jag2 | Hes1 | Hes2 | Hes5 | Hes6 | Hey1 | Hey2 |
| --- | --- | --- | --- | --- | --- | --- | --- | --- | --- | --- | --- | --- | --- | --- | --- | --- | --- | --- | --- | --- | --- | --- |
| GBM1 | 0.8 | 1.4 | 1.1 | 0.0 | 0.5 | 0.6 | **56.8** | 0.4 | 0.0 | **2.5** | 0.0 | **2.6** | 0.0 | 0.0 | 0.0 | 0.0 | 0.5 | 0.0 | 0.0 | 0.0 | 0.0 | 0.3 |
| GBM2 | **5.4** | **15.1** | **19.9** | 0.1 | 0.6 | 0.0 | 0.0 | 0.6 | 0.0 | **34.0** | 0.0 | **5.2** | 0.0 | **7.8** | 0.0 | **5.7** | **3.0** | 0.0 | 0.0 | 0.0 | **2.9** | **6.6** |
| GBM3 | 0.0 | **10.5** | **24.7** | 0.0 | **6.7** | 0.0 | 0.0 | 0.0 | 0.0 | 0.0 | 0.0 | 0.0 | 0.0 | 0.0 | 0.0 | **21.6** | **45.3** | 0.0 | 0.0 | 0.0 | 1.1 | **4.3** |
| GBM4 | **4.3** | **2.8** | 1.3 | 0.0 | **8.6** | 0.0 | 0.0 | 0.7 | **29.4** | **105.2** | 0.0 | 0.0 | 0.0 | 0.0 | 0.0 | **19.3** | **56.6** | 0.0 | 0.0 | **5.2** | 0.4 | **11.4** |
| GBM5 | 0.5 | **2.5** | 0.4 | 0.0 | **5.1** | 0.3 | **6.9** | **2.9** | **11.6** | **7.4** | 0.0 | **52.0** | 0.6 | **2.1** | **33.4** | 0.8 | 0.3 | 0.0 | 0.0 | 0.8 | 0.2 | 0.3 |
| GBM6 | 0.8 | **15.9** | **63.3** | **1.9** | **3.8** | **3.1** | **60.5** | 0.3 | **2.1** | 0.4 | 0.0 | 0.6 | 0.0 | 0.3 | **1.5** | 0.0 | 1.3 | 0.0 | 0.0 | 0.3 | 0.4 | 0.7 |
| GBM7 | **76.7** | **17.9** | **12.8** | 0.3 | **166.8** | 0.2 | 0.0 | 0.3 | 0.5 | **1.5** | **6.5** | **19.7** | 0.7 | 1.2 | **4.8** | 0.1 | **23.7** | 0.0 | 0.1 | **2.9** | **7.1** | 0.6 |
| GBM8 | **46.4** | **50.2** | **52.3** | 0.1 | **309.5** | 0.1 | **7.3** | 0.2 | 0.1 | 0.3 | **10.2** | **34.9** | **82.3** | 0.4 | **2.8** | 0.0 | **18.5** | 0.0 | 0.0 | **13.3** | **10.7** | **15.4** |
| GBM9 | **10.6** | **3.2** | **7.9** | 0.1 | 1.3 | **5.8** | 0.3 | **3.7** | 1.4 | 0.0 | 0.0 | **2.0** | 0.5 | **10.8** | **277.6** | 0.5 | **9.8** | 0.0 | 0.1 | 0.0 | 0.9 | **4.3** |
| GBM10 | **42.3** | **30.8** | **75.5** | 0.1 | **94.8** | 0.4 | **20.0** | **12.2** | **1.9** | 0.1 | **107.5** | **281.4** | **26.8** | **8.3** | **40.3** | 1.1 | **52.4** | 0.0 | 0.2 | **54.3** | **25.6** | 0.6 |
| GBM11 | **12.8** | **93.9** | **368.0** | 0.2 | **134.9** | 0.6 | **361.3** | **8.6** | 0.3 | 0.1 | **13.8** | **61.9** | **6.2** | **1.5** | **16.8** | 0.5 | **39.8** | **30.6** | 0.0 | **29.7** | **8.0** | **2.0** |
| GBM12 | 0.0 | 0.9 | **15.5** | 0.0 | 1.3 | 0.0 | **22.9** | 0.0 | 0.0 | 0.0 | 0.0 | 0.0 | 0.0 | 0.0 | 0.0 | 0.0 | 0.0 | 0.0 | 0.0 | 0.0 | 0.1 | 0.2 |
| GBM13 | 0.0 | **1.7** | 0.1 | 0.0 | 0.0 | 0.0 | **14.5** | 0.0 | 0.0 | 0.0 | 0.0 | 0.0 | 0.0 | 0.5 | 0.0 | 0.0 | 0.1 | 0.0 | 0.0 | 0.0 | 0.0 | 0.0 |
| GBM14 | **1.5** | **3.6** | 0.4 | 0.0 | 0.1 | 0.1 | **2.6** | 0.6 | 1.2 | 1.1 | 0.0 | **21.9** | **6.2** | 0.1 | **3.7** | 0.4 | 0.2 | **99.9** | 0.3 | **2.8** | **1.6** | 0.9 |
| GBM15 | 0.6 | 0.7 | **21.8** | 0.0 | 1.1 | 0.4 | **6.4** | 1.1 | 0.6 | **2.5** | 0.5 | **1.7** | 0.1 | 1.3 | **41.1** | 0.1 | 0.3 | **4.3** | 0.0 | 0.1 | 0.3 | 0.4 |
| GBM16 | **4.0** | **8.6** | **7.5** | 0.1 | **4.5** | 0.4 | **24.6** | **3.0** | **2.3** | **1.8** | 1.1 | **20.1** | 1.2 | 0.7 | **94.9** | 0.1 | 0.5 | **40.9** | 0.1 | 0.3 | 1.2 | 0.9 |
| GBM17 | **5.0** | **4.9** | **4.0** | 0.1 | **3.5** | 0.1 | **6.8** | **1.7** | 0.8 | 0.6 | 0.0 | **23.6** | **4.6** | 0.3 | **44.9** | 0.1 | 0.1 | 0.0 | 0.2 | 0.4 | 1.1 | **1.5** |
| GBM18 | **3.0** | **12.2** | 1.3 | 0.1 | 0.6 | 0.0 | **1.6** | **1.6** | **1.6** | **4.5** | 0.0 | **135.5** | **15.0** | 0.1 | **32.2** | 0.1 | 0.8 | 0.0 | **5.3** | **2.7** | **15.2** | **3.5** |
| GBM19 | **2.2** | **10.2** | **2.1** | 0.1 | **3.8** | 0.1 | 0.3 | **18.6** | **2.4** | 0.5 | 0.0 | **68.7** | 0.9 | 0.7 | **103.8** | 0.2 | **3.1** | 0.9 | **2.1** | **2.4** | **17.6** | 1.0 |
| GBM20 | **4.2** | **42.3** | **50.4** | 0.3 | **49.4** | 0.2 | **2862.3** | **4.2** | **1.9** | **2.1** | 0.0 | 1.1 | 0.0 | 0.3 | **53.9** | 0.2 | 1.3 | **2.8** | 0.0 | 0.0 | **3.9** | 0.2 |
| GBM21 | **1.8** | **2.8** | **9.6** | 0.1 | **7.3** | 0.0 | **41.9** | 0.2 | 0.4 | 1.1 | 0.0 | 0.2 | 0.0 | 0.2 | **13.7** | 0.0 | 0.2 | 0.2 | 0.0 | 0.0 | 0.3 | 0.0 |
| GBM22 | 0.4 | **2.1** | 0.6 | 0.1 | **2.3** | 0.1 | **2.7** | 0.3 | 0.3 | 0.2 | 0.0 | **11.9** | 0.1 | 0.3 | **5.1** | 0.4 | 0.1 | 0.0 | 0.0 | 0.3 | 0.1 | 0.1 |
| GBM23 | 0.0 | 0.0 | 0.1 | 0.0 | 0.2 | 0.0 | 0.0 | 0.0 | 0.0 | 0.2 | 0.0 | 0.3 | 0.0 | 0.0 | 0.0 | 0.0 | 0.0 | 0.0 | 0.0 | 0.0 | 0.0 | 0.0 |
| GBM24 | **1.8** | **15.0** | **96.7** | 0.2 | **6.7** | 0.5 | **265.3** | 0.3 | 1.4 | 0.3 | 0.0 | **2.7** | 0.0 | 0.7 | **5.0** | 0.3 | 0.2 | 0.6 | 0.0 | 0.2 | **4.8** | 0.3 |
| GBM25 | 1.0 | **1.7** | **1.6** | 0.0 | **8.8** | 0.0 | **87.5** | 0.0 | 0.2 | 0.1 | 0.0 | 0.2 | 0.0 | 0.3 | 0.0 | 0.1 | 0.1 | 0.0 | 0.0 | 0.0 | 0.4 | 0.0 |
| GBM26 | 0.0 | 0.1 | 0.3 | 0.0 | 0.1 | 0.0 | 1.1 | 0.0 | 0.0 | 0.0 | 0.0 | 0.0 | 0.0 | 0.1 | 0.0 | 0.0 | 0.0 | 0.0 | 0.0 | 0.0 | 0.0 | 0.0 |
| GBM27 | **2.7** | **13.6** | **69.5** | 0.1 | **2.8** | 0.1 | **451.1** | **1.6** | 1.3 | 0.6 | 0.0 | **4.2** | 0.0 | **5.5** | **3.0** | 0.7 | **1.7** | 0.8 | 0.0 | 0.1 | **1.5** | 0.3 |
| GBM28 | 0.0 | 1.3 | **61.9** | 0.1 | **2.4** | 0.1 | **101.6** | 0.2 | 0.2 | 0.2 | 0.0 | 1.3 | 0.0 | 0.5 | **8.4** | 0.1 | 0.4 | **2.5** | 0.0 | 0.0 | 0.1 | 0.1 |
| GBM29 | 0.6 | **2.5** | **9.2** | 0.0 | **19.2** | 0.1 | **9.7** | **1.5** | 0.2 | 0.5 | 0.0 | **6.6** | 0.3 | 0.7 | **8.8** | 0.0 | **2.5** | 0.5 | 0.0 | 0.3 | 0.1 | 0.6 |
| GBM30 | 0.9 | **11.2** | **79.8** | 0.1 | **7.5** | **5.9** | **8.3** | **4.3** | **1.9** | **4.2** | 0.0 | **3.0** | 1.2 | 0.8 | 0.0 | 0.7 | **1.9** | **27.7** | 0.2 | **3.0** | 0.5 | 0.2 |
| GBM31 | **3.0** | **10.9** | **125.7** | 0.2 | **2.9** | 0.2 | **537.2** | **7.2** | **2.0** | **3.3** | 1.3 | **35.4** | 0.8 | **1.9** | **131.0** | 0.6 | 0.8 | 0.0 | 0.5 | **6.8** | **10.0** | **1.6** |
| GBM32 | 0.7 | **3.2** | **10.5** | 0.0 | **27.1** | 0.1 | **40.4** | 0.8 | 0.7 | **2.1** | 0.0 | **2.7** | 0.2 | 0.9 | **885.9** | 0.4 | **1.5** | 0.0 | 0.0 | 0.2 | 0.6 | 0.2 |
| GBM33 | **3.6** | **5.7** | **6.1** | 0.2 | **7.2** | 0.2 | **3.8** | **3.2** | **1.6** | **3.8** | 0.0 | **34.0** | 0.9 | 0.4 | **158.4** | 0.5 | 1.1 | 0.0 | 0.7 | **6.8** | **5.9** | **1.8** |
| GBM34 | 1.4 | **13.0** | **10.1** | 0.2 | **5.6** | 0.0 | **3.6** | 0.5 | 0.9 | **1.9** | 0.0 | **5.4** | 0.0 | 1.3 | **78.3** | 0.7 | **2.4** | 0.0 | 0.0 | 1.1 | 0.2 | 0.2 |
| GBM35 | **26.8** | **107.3** | **372.1** | 1.2 | **120.4** | 0.1 | **3118.4** | **2.0** | **2.5** | **4.1** | 0.0 | **52.1** | **1.6** | **2.0** | **895.3** | 1.4 | **2.9** | **52.5** | 0.0 | **3.2** | **5.0** | 1.0 |
| % | 54 | 83 | 74 | 3 | 71 | 9 | 77 | 43 | 34 | 43 | 11 | 69 | 20 | 23 | 71 | 9 | 43 | 23 | 6 | 34 | 40 | 29 |

Note: mRNA expression of all genes is relative to 18S rRNA and normalized to normal brain levels. Expression ≥1.5-fold has been marked in bold to indicate significant upregulation relative to normal brain. The last row shows the percentage of samples with significant gene upregulation.
