# Supplementary material for: A Combined Gene Signature of Hypoxia and Notch Pathway in Human Glioblastoma and Its Prognostic Relevance
Source: PLoS One. 2015 Mar 3;10(3):e0118201. doi: 10.1371/journal.pone.0118201 (PMC4348203; doi:10.1371/journal.pone.0118201)
Supplement: S4 Table — (DOC) [file pone.0118201.s010.doc]

**Table S4.** Expression values of hypoxia markers in 35 GBM samples grouped by tertiles in decreasing order of HIF-1α expression

| **Tertile** | **Rank** | **Samples** | **HIF-1α** | PGK1 | VEGF | OPN | CA9 | EPO |
| --- | --- | --- | --- | --- | --- | --- | --- | --- |
| **HIGH HIF-1α TERTILE** | **1** | **GBM7** | **76.7** | 17.9 | 12.8 | 166.8 | 0.0 | 0.2 |
|  | **2** | **GBM8** | **46.4** | 50.2 | 52.3 | 309.5 | 7.3 | 0.1 |
|  | **3** | **GBM10** | **42.3** | 30.8 | 75.5 | 94.8 | 20.0 | 0.4 |
|  | **4** | **GBM35** | **26.8** | 107.3 | 372.1 | 120.4 | 3118.4 | 0.1 |
|  | **5** | **GBM11** | **12.8** | 93.9 | 368.0 | 134.9 | 361.3 | 0.6 |
|  | **6** | **GBM9** | **10.6** | 3.2 | 7.9 | 1.3 | 0.3 | 5.8 |
|  | **7** | **GBM2** | **5.4** | 15.1 | 19.9 | 0.6 | 0.0 | 0.0 |
|  | **8** | **GBM17** | **5.0** | 4.9 | 4.0 | 3.5 | 6.8 | 0.1 |
|  | **9** | **GBM4** | **4.3** | 2.8 | 1.3 | 8.6 | 0.0 | 0.0 |
|  | **10** | **GBM20** | **4.2** | 42.3 | 50.4 | 49.4 | 2862.3 | 0.2 |
|  | **11** | **GBM16** | **4.0** | 8.6 | 7.5 | 4.5 | 24.6 | 0.4 |
|  | **12** | **GBM33** | **3.6** | 5.7 | 6.1 | 7.2 | 3.8 | 0.2 |
|  |  |  |  |  |  |  |  |  |
| **INTERMEDIATE HIF-1α TERTILE** | **13** | **GBM31** | **3.0** | 10.9 | 125.7 | 2.9 | 537.2 | 0.2 |
|  | **14** | **GBM18** | **3.0** | 12.2 | 1.3 | 0.6 | 1.6 | 0.0 |
|  | **15** | **GBM27** | **2.7** | 13.6 | 69.5 | 2.8 | 451.1 | 0.1 |
|  | **16** | **GBM19** | **2.2** | 10.2 | 2.1 | 3.8 | 0.3 | 0.1 |
|  | **17** | **GBM24** | **1.8** | 15.0 | 96.7 | 6.7 | 265.3 | 0.5 |
|  | **18** | **GBM21** | **1.8** | 2.8 | 9.6 | 7.3 | 41.9 | 0.0 |
|  | **19** | **GBM14** | **1.5** | 3.6 | 0.4 | 0.1 | 2.6 | 0.1 |
|  | **20** | **GBM34** | **1.4** | 13.0 | 10.1 | 5.6 | 3.6 | 0.0 |
|  | **21** | **GBM25** | **1.0** | 1.7 | 1.6 | 8.8 | 87.5 | 0.0 |
|  | **22** | **GBM30** | **0.9** | 11.2 | 79.8 | 7.5 | 8.3 | 5.9 |
|  | **23** | **GBM6** | **0.8** | 15.9 | 63.3 | 3.8 | 60.5 | 3.1 |
|  |  |  |  |  |  |  |  |  |
| **LOW HIF-1α TERTILE** | **24** | **GBM1** | **0.8** | 1.4 | 1.1 | 0.5 | 56.8 | 0.6 |
|  | **25** | **GBM32** | **0.7** | 3.2 | 10.5 | 27.1 | 40.4 | 0.1 |
|  | **26** | **GBM15** | **0.6** | 0.7 | 21.8 | 1.1 | 6.4 | 0.4 |
|  | **27** | **GBM29** | **0.6** | 2.5 | 9.2 | 19.2 | 9.7 | 0.1 |
|  | **28** | **GBM5** | **0.5** | 2.5 | 0.4 | 5.1 | 6.9 | 0.3 |
|  | **29** | **GBM22** | **0.4** | 2.1 | 0.6 | 2.3 | 2.7 | 0.1 |
|  | **30** | **GBM26** | **0.0** | 0.1 | 0.3 | 0.1 | 1.1 | 0.0 |
|  | **31** | **GBM3** | **0.0** | 10.5 | 24.7 | 6.7 | 0.0 | 0.0 |
|  | **32** | **GBM12** | **0.0** | 0.9 | 15.5 | 1.3 | 22.9 | 0.0 |
|  | **33** | **GBM13** | **0.0** | 1.7 | 0.1 | 0.0 | 14.5 | 0.0 |
|  | **34** | **GBM23** | **0.0** | 0.0 | 0.1 | 0.2 | 0.0 | 0.0 |
|  | **35** | **GBM28** | **0.0** | 1.3 | 61.9 | 2.4 | 101.6 | 0.1 |
|  |  |  |  |  |  |  |  |  |
|  |  | p-value | 0.007* | 0.008* | 0.054* | 0.014* | 0.076 | 0.142 |

(*) Significant difference (p ≤ 0.05) in gene expression across high and low tertiles
