# Supplementary material for: A Combined Gene Signature of Hypoxia and Notch Pathway in Human Glioblastoma and Its Prognostic Relevance
Source: PLoS One. 2015 Mar 3;10(3):e0118201. doi: 10.1371/journal.pone.0118201 (PMC4348203; doi:10.1371/journal.pone.0118201)
Supplement: S7 Table — (DOC) [file pone.0118201.s013.doc]

**Table S7.** Expression values of Notch signaling genes in 35 GBM samples grouped by tertiles in decreasing order of OPN expression

| **Tertile** | **Rank** | **Samples** | **OPN** | Notch1 | Notch2 | Notch3 | Notch4 | Dll1 | Dll3 | Dll4 | Jag1 | Jag2 | Hes1 | Hes2 | Hes5 | Hes6 | Hey1 | Hey2 |
| --- | --- | --- | --- | --- | --- | --- | --- | --- | --- | --- | --- | --- | --- | --- | --- | --- | --- | --- |
| **HIGH OPN TERTILE** | **1** | **GBM8** | **309.5** | 0.2 | 0.1 | 0.3 | 10.2 | 34.9 | 82.3 | 0.4 | 2.8 | 0.0 | 18.5 | 0.0 | 0.0 | 13.3 | 10.7 | 15.4 |
|  | **2** | **GBM7** | **166.8** | 0.3 | 0.5 | 1.5 | 6.5 | 19.7 | 0.7 | 1.2 | 4.8 | 0.1 | 23.7 | 0.0 | 0.1 | 2.9 | 7.1 | 0.6 |
|  | **3** | **GBM11** | **134.9** | 8.6 | 0.3 | 0.1 | 13.8 | 61.9 | 6.2 | 1.5 | 16.8 | 0.5 | 39.8 | 30.6 | 0.0 | 29.7 | 8.0 | 2.0 |
|  | **4** | **GBM35** | **120.4** | 2.0 | 2.5 | 4.1 | 0.0 | 52.1 | 1.6 | 2.0 | 895.3 | 1.4 | 2.9 | 52.5 | 0.0 | 3.2 | 5.0 | 1.0 |
|  | **5** | **GBM10** | **94.8** | 12.2 | 1.9 | 0.1 | 107.5 | 281.4 | 26.8 | 8.3 | 40.3 | 1.1 | 52.4 | 0.0 | 0.2 | 54.3 | 25.6 | 0.6 |
|  | **6** | **GBM20** | **49.4** | 4.2 | 1.9 | 2.1 | 0.0 | 1.1 | 0.0 | 0.3 | 53.9 | 0.2 | 1.3 | 2.8 | 0.0 | 0.0 | 3.9 | 0.2 |
|  | **7** | **GBM32** | **27.1** | 0.8 | 0.7 | 2.1 | 0.0 | 2.7 | 0.2 | 0.9 | 885.9 | 0.4 | 1.5 | 0.0 | 0.0 | 0.2 | 0.6 | 0.2 |
|  | **8** | **GBM29** | **19.2** | 1.5 | 0.2 | 0.5 | 0.0 | 6.6 | 0.3 | 0.7 | 8.8 | 0.0 | 2.5 | 0.5 | 0.0 | 0.3 | 0.1 | 0.6 |
|  | **9** | **GBM25** | **8.8** | 0.0 | 0.2 | 0.1 | 0.0 | 0.2 | 0.0 | 0.3 | 0.0 | 0.1 | 0.1 | 0.0 | 0.0 | 0.0 | 0.4 | 0.0 |
|  | **10** | **GBM4** | **8.6** | 0.7 | 29.4 | 105.2 | 0.0 | 0.0 | 0.0 | 0.0 | 0.0 | 19.3 | 56.6 | 0.0 | 0.0 | 5.2 | 0.4 | 11.4 |
|  | **11** | **GBM30** | **7.5** | 4.3 | 1.9 | 4.2 | 0.0 | 3.0 | 1.2 | 0.8 | 0.0 | 0.7 | 1.9 | 27.7 | 0.2 | 3.0 | 0.5 | 0.2 |
|  | **12** | **GBM21** | **7.3** | 0.2 | 0.4 | 1.1 | 0.0 | 0.2 | 0.0 | 0.2 | 13.7 | 0.0 | 0.2 | 0.2 | 0.0 | 0.0 | 0.3 | 0.0 |
|  |  |  |  |  |  |  |  |  |  |  |  |  |  |  |  |  |  |  |
| **INTERMEDIATE OPN TERTILE** | **13** | **GBM33** | **7.2** | 3.2 | 1.6 | 3.8 | 0.0 | 34.0 | 0.9 | 0.4 | 158.4 | 0.5 | 1.1 | 0.0 | 0.7 | 6.8 | 5.9 | 1.8 |
|  | **14** | **GBM24** | **6.7** | 0.3 | 1.4 | 0.3 | 0.0 | 2.7 | 0.0 | 0.7 | 5.0 | 0.3 | 0.2 | 0.6 | 0.0 | 0.2 | 4.8 | 0.3 |
|  | **15** | **GBM3** | **6.7** | 0.0 | 0.0 | 0.0 | 0.0 | 0.0 | 0.0 | 0.0 | 0.0 | 21.6 | 45.3 | 0.0 | 0.0 | 0.0 | 1.1 | 4.3 |
|  | **16** | **GBM34** | **5.6** | 0.5 | 0.9 | 1.9 | 0.0 | 5.4 | 0.0 | 1.3 | 78.3 | 0.7 | 2.4 | 0.0 | 0.0 | 1.1 | 0.2 | 0.2 |
|  | **17** | **GBM5** | **5.1** | 2.9 | 11.6 | 7.4 | 0.0 | 52.0 | 0.6 | 2.1 | 33.4 | 0.8 | 0.3 | 0.0 | 0.0 | 0.8 | 0.2 | 0.3 |
|  | **18** | **GBM16** | **4.5** | 3.0 | 2.3 | 1.8 | 1.1 | 20.1 | 1.2 | 0.7 | 94.9 | 0.1 | 0.5 | 40.9 | 0.1 | 0.3 | 1.2 | 0.9 |
|  | **19** | **GBM6** | **3.8** | 0.3 | 2.1 | 0.4 | 0.0 | 0.6 | 0.0 | 0.3 | 1.5 | 0.0 | 1.3 | 0.0 | 0.0 | 0.3 | 0.4 | 0.7 |
|  | **20** | **GBM19** | **3.8** | 18.6 | 2.4 | 0.5 | 0.0 | 68.7 | 0.9 | 0.7 | 103.8 | 0.2 | 3.1 | 0.9 | 2.1 | 2.4 | 17.6 | 1.0 |
|  | **21** | **GBM17** | **3.5** | 1.7 | 0.8 | 0.6 | 0.0 | 23.6 | 4.6 | 0.3 | 44.9 | 0.1 | 0.1 | 0.0 | 0.2 | 0.4 | 1.1 | 1.5 |
|  | **22** | **GBM31** | **2.9** | 7.2 | 2.0 | 3.3 | 1.3 | 35.4 | 0.8 | 1.9 | 131.0 | 0.6 | 0.8 | 0.0 | 0.5 | 6.8 | 10.0 | 1.6 |
|  | **23** | **GBM27** | **2.8** | 1.6 | 1.3 | 0.6 | 0.0 | 4.2 | 0.0 | 5.5 | 3.0 | 0.7 | 1.7 | 0.8 | 0.0 | 0.1 | 1.5 | 0.3 |
|  |  |  |  |  |  |  |  |  |  |  |  |  |  |  |  |  |  |  |
| **LOW OPN TERTILE** | **24** | **GBM28** | **2.4** | 0.2 | 0.2 | 0.2 | 0.0 | 1.3 | 0.0 | 0.5 | 8.4 | 0.1 | 0.4 | 2.5 | 0.0 | 0.0 | 0.1 | 0.1 |
|  | **25** | **GBM22** | **2.3** | 0.3 | 0.3 | 0.2 | 0.0 | 11.9 | 0.1 | 0.3 | 5.1 | 0.4 | 0.1 | 0.0 | 0.0 | 0.3 | 0.1 | 0.1 |
|  | **26** | **GBM12** | **1.3** | 0.0 | 0.0 | 0.0 | 0.0 | 0.0 | 0.0 | 0.0 | 0.0 | 0.0 | 0.0 | 0.0 | 0.0 | 0.0 | 0.1 | 0.2 |
|  | **27** | **GBM9** | **1.3** | 3.7 | 1.4 | 0.0 | 0.0 | 2.0 | 0.5 | 10.8 | 277.6 | 0.5 | 9.8 | 0.0 | 0.1 | 0.0 | 0.9 | 4.3 |
|  | **28** | **GBM15** | **1.1** | 1.1 | 0.6 | 2.5 | 0.5 | 1.7 | 0.1 | 1.3 | 41.1 | 0.1 | 0.3 | 4.3 | 0.0 | 0.1 | 0.3 | 0.4 |
|  | **29** | **GBM18** | **0.6** | 1.6 | 1.6 | 4.5 | 0.0 | 135.5 | 15.0 | 0.1 | 32.2 | 0.1 | 0.8 | 0.0 | 5.3 | 2.7 | 15.2 | 3.5 |
|  | **30** | **GBM2** | **0.6** | 0.6 | 0.0 | 34.0 | 0.0 | 5.2 | 0.0 | 7.8 | 0.0 | 5.7 | 3.0 | 0.0 | 0.0 | 0.0 | 2.9 | 6.6 |
|  | **31** | **GBM1** | **0.5** | 0.4 | 0.0 | 2.5 | 0.0 | 2.6 | 0.0 | 0.0 | 0.0 | 0.0 | 0.5 | 0.0 | 0.0 | 0.0 | 0.0 | 0.3 |
|  | **32** | **GBM23** | **0.2** | 0.0 | 0.0 | 0.2 | 0.0 | 0.3 | 0.0 | 0.0 | 0.0 | 0.0 | 0.0 | 0.0 | 0.0 | 0.0 | 0.0 | 0.0 |
|  | **33** | **GBM14** | **0.1** | 0.6 | 1.2 | 1.1 | 0.0 | 21.9 | 6.2 | 0.1 | 3.7 | 0.4 | 0.2 | 99.9 | 0.3 | 2.8 | 1.6 | 0.9 |
|  | **34** | **GBM26** | **0.1** | 0.0 | 0.0 | 0.0 | 0.0 | 0.0 | 0.0 | 0.1 | 0.0 | 0.0 | 0.0 | 0.0 | 0.0 | 0.0 | 0.0 | 0.0 |
|  | **35** | **GBM13** | **0.0** | 0.0 | 0.0 | 0.0 | 0.0 | 0.0 | 0.0 | 0.5 | 0.0 | 0.0 | 0.1 | 0.0 | 0.0 | 0.0 | 0.0 | 0.0 |
|  |  |  |  |  |  |  |  |  |  |  |  |  |  |  |  |  |  |  |
|  |  | p-value | 0.006* | 0.040* | 0.127 | 0.248 | 0.111 | 0.186 | 0.136 | 0.371 | 0.112 | 0.209 | 0.015* | 0.474 | 0.170 | 0.046* | 0.091 | 0.212 |

(*) Significant difference (p ≤ 0.05) in gene expression across high and low tertiles
