# Supplementary material for: A Combined Gene Signature of Hypoxia and Notch Pathway in Human Glioblastoma and Its Prognostic Relevance
Source: PLoS One. 2015 Mar 3;10(3):e0118201. doi: 10.1371/journal.pone.0118201 (PMC4348203; doi:10.1371/journal.pone.0118201)
Supplement: S10 Table — (DOC) [file pone.0118201.s016.doc]

**Table S10.** Summary of sensitivity and specificity of CA9 and VEGF expression in diagnosis of Notch genes’ overexpression in 35 GBM tumors

| Sensitivity and specificity of CA9 expression: | | | | | | | |
| --- | --- | --- | --- | --- | --- | --- | --- |
| Variable  (transformed on basis of CA9) | Count | | | PPV  (%) | Sensitivity  (%) | NPV  (%) | Specificity  (%) |
|  |  | CA9 (predictor) | |  |  |  |  |
|  |  | 1 | 0 |  |  |  |  |
| Notch1 | 1 | 2 | 1 | 66.7 | 11.1 | 50.0 | **94.1** |
| Notch2 | 1 | 0 | 2 | 0 | 0 | 45.5 | **88.2** |
| Notch3 | 1 | 0 | 2 | 0 | 0 | 45.5 | **88.2** |
| Notch4 | 1 | 2 | 1 | 66.7 | 11.1 | 50.0 | **94.1** |
| Dll1 | 1 | 5 | 9 | 35.7 | 27.8 | 38.1 | 47.1 |
| Dll3 | 1 | 1 | 2 | 33.3 | 5.6 | 46.9 | **88.2** |
| Dll4 | 1 | 1 | 1 | 50.0 | 5.6 | 48.5 | **94.1** |
| Jag1 | 1 | 10 | 8 | 55.6 | **55.6** | 52.9 | **52.9** |
| Jag2 | 1 | 0 | 2 | 0 | 0 | 45.5 | **88.2** |
| Hes1 | 1 | 2 | 5 | 28.6 | 11.1 | 42.9 | 70.6 |
| Hes2 | 1 | 4 | 1 | 80.0 | 22.2 | 53.3 | **94.1** |
| Hes5 | 1 |  |  |  |  | 48.6 | **100.0** |
| Hes6 | 1 | 2 | 1 | 66.7 | 11.1 | 50.0 | **94.1** |
| Hey1 | 1 | 2 | 3 | 40.0 | 11.1 | 46.7 | **82.4** |
| Hey2 | 1 | 0 | 2 | 0 | 0 | 45.5 | **88.2** |
| Sensitivity and specificity of VEGF expression: | | | | | | | |
| Variable  (transformed on basis of VEGF) | Count | | | PPV  (%) | Sensitivity  (%) | NPV  (%) | Specificity  (%) |
|  |  | VEGF (predictor) | |  |  |  |  |
|  |  | 1 | 0 |  |  |  |  |
| Notch1 | 1 | 1 | 1 | 50.0 | 5.9 | 51.5 | **94.4** |
| Notch2 | 1 | 0 | 2 | 0 | 0 | 48.5 | **88.9** |
| Notch3 | 1 | 1 | 1 | 50.0 | 5.9 | 51.5 | **94.4** |
| Notch4 | 1 | 3 | 0 | 100.0 | 17.6 | 56.3 | **100.0** |
| Dll1 | 1 | 6 | 8 | 42.9 | 35.3 | 47.6 | **55.6** |
| Dll3 | 1 | 2 | 1 | 66.7 | 11.8 | 53.1 | **94.4** |
| Dll4 | 1 | 0 | 1 | 0 | 0 | 50.0 | **94.4** |
| Jag1 | 1 | 7 | 9 | 43.8 | 41.2 | 47.4 | **50.0** |
| Jag2 | 1 | 1 | 1 | 50.0 | 5.9 | 51.5 | **94.4** |
| Hes1 | 1 | 5 | 1 | 83.3 | 29.4 | 58.6 | **94.4** |
| Hes2 | 1 | 3 | 2 | 60.0 | 17.6 | 53.3 | **88.9** |
| Hes5 | 1 |  |  |  |  | 51.4 | **100.0** |
| Hes6 | 1 | 3 | 0 | 100.0 | 17.6 | 56.3 | **100.0** |
| Hey1 | 1 | 2 | 2 | 50.0 | 11.8 | 51.6 | **88.9** |
| Hey2 | 1 | 1 | 1 | 50.0 | 5.9 | 51.5 | **94.4** |

Note: Gene expression values were transformed into binary data where 1 denotes values ≥ CA9 or VEGF median expression and 0 denotes values ≤ CA9 or VEGF expression. Sensitivity and specificity ≥ 50% were taken as cut-offs for predictive value of CA9 or VEGF and have been indicated in bold.

Abbreviations: PPV, positive predictive value; NPV, negative predictive value
