# Supplementary material for: A Combined Gene Signature of Hypoxia and Notch Pathway in Human Glioblastoma and Its Prognostic Relevance
Source: PLoS One. 2015 Mar 3;10(3):e0118201. doi: 10.1371/journal.pone.0118201 (PMC4348203; doi:10.1371/journal.pone.0118201)
Supplement: S11 Table — (DOC) [file pone.0118201.s017.doc]

**Table S11.** Summary of results of linear regression analysis using all possible combinations of hypoxia markers (parts (i)-(xiii)) as independent variables for their combined association with the expression level of individual Notch genes

**(i) Predictor: HI**F-1α, PGK1

| **Notch signaling gene**  **(Dependent variable)** | **r** | **r^2^** | **p-value of regression model** | **β** | |
| --- | --- | --- | --- | --- | --- |
| Notch4 | 0.46 | 0.21 | 0.022 | HIF-1α  PGK1 | 0.51  0.01 |
| Dll1 | 0.42 | 0.18 | 0.045 | HIF-1α  PGK1 | 1.08  0.33 |
| Dll3 | 0.52 | 0.27 | 0.006 | HIF-1α  PGK1 | 0.41  0.07 |
| Hes1 | 0.45 | 0.21 | 0.025 | HIF-1α  PGK1 | 0.37  0.09 |
| Hes6 | 0.54 | 0.29 | 0.005 | HIF-1α  PGK1 | 0.22  0.13 |
| Hey1 | 0.52 | 0.27 | 0.007 | HIF-1α  PGK1 | 0.15  0.04 |

**(ii)** Predictor: HIF-1α, VEGF

| **Notch signaling gene**  **(Dependent variable)** | **r** | **r^2^** | **p-value of regression model** | **β** | |
| --- | --- | --- | --- | --- | --- |
| Notch4 | 0.46 | 0.21 | 0.022 | HIF-1α  VEGF | 0.51  0.01 |
| Dll1 | 0.42 | 0.17 | 0.049 | HIF-1α  VEGF | 1.21  0.07 |
| Dll3 | 0.51 | 0.26 | 0.008 | HIF-1α  VEGF | 0.47  -0.01 |
| Hes1 | 0.46 | 0.21 | 0.024 | HIF-1α  VEGF | 0.40  0.02 |
| Hes6 | 0.55 | 0.30 | 0.003 | HIF-1α  VEGF | 0.25  0.04 |
| Hey1 | 0.51 | 0.26 | 0.009 | HIF-1α  VEGF | 0.17  0.01 |

**(iii)** Predictor: HIF-1α, OPN

| **Notch signaling gene**  **(Dependent variable)** | **r** | **r^2^** | **p-value of regression model** | **β** | |
| --- | --- | --- | --- | --- | --- |
| Notch4 | 0.47 | 0.23 | 0.017 | HIF-1α  OPN | 0.70  -0.06 |
| Dll1 | 0.41 | 0.17 | 0.054 | HIF-1α  OPN | 1.72  -0.13 |
| Dll3 | 0.82 | 0.67 | 0.000 | HIF-1α  OPN | -0.34  0.25 |
| Hes1 | 0.44 | 0.19 | 0.032 | HIF-1α  OPN | 0.34  0.03 |
| Hes6 | 0.49 | 0.24 | 0.014 | HIF-1α  OPN | 0.17  0.04 |
| Hey1 | 0.49 | 0.24 | 0.012 | HIF-1α  OPN | 0.17  0.004 |
| Hey2 | 0.56 | 0.32 | 0.002 | HIF-1α  OPN | -0.07  0.04 |

**(iv)** Predictor: HIF-1α, CA9

| **Notch signaling gene**  **(Dependent variable)** | **r** | **r^2^** | **p-value of regression model** | **β** | |
| --- | --- | --- | --- | --- | --- |
| Notch4 | 0.47 | 0.22 | 0.018 | HIF-1α  CA9 | 0.53  -0.003 |
| Dll3 | 0.53 | 0.28 | 0.006 | HIF-1α  CA9 | 0.47  -0.003 |
| J1 | 0.48 | 0.23 | 0.016 | HIF-1α  CA9 | 0.43  0.14 |
| Hes1 | 0.46 | 0.21 | 0.023 | HIF-1α  CA9 | 0.44  -0.003 |
| Hes6 | 0.47 | 0.22 | 0.018 | HIF-1α  CA9 | 0.30  -0.001 |
| Hey1 | 0.49 | 0.24 | 0.012 | HIF-1α  CA9 | 0.18  1.6E-05 |

**(v) Predictor: PGK1, VEGF**

| **Notch signaling gene**  **(Dependent variable)** | **r** | **r^2^** | **p-value of regression model** | **β** | |
| --- | --- | --- | --- | --- | --- |
| Dll3 | 0.60 | 0.36 | 0.001 | PGK1  VEGF | 0.82  -0.19 |
| Hes6 | 0.44 | 0.19 | 0.033 | PGK1  VEGF | 0.20  -0.004 |
| Hey1 | 0.41 | 0.17 | 0.052 | PGK1  VEGF | 0.19  -0.03 |
| Hey2 | 0.46 | 0.21 | 0.021 | PGK1  VEGF | 0.14  -0.04 |

**(vi) Predictor:** PGK1, OPN

| **Notch signaling gene**  **(Dependent variable)** | **r** | **r^2^** | **p-value of regression model** | **β** | |
| --- | --- | --- | --- | --- | --- |
| Dll3 | 0.83 | 0.69 | 0.000 | PGK1  OPN | -0.21  0.23 |
| J1 | 0.42 | 0.18 | 0.042 | PGK1  OPN | 4.62  -0.72 |
| Hes6 | 0.49 | 0.24 | 0.012 | PGK1  OPN | 0.10  0.05 |
| Hey1 | 0.43 | 0.18 | 0.040 | PGK1  OPN | 0.03  0.03 |
| Hey2 | 0.56 | 0.31 | 0.003 | PGK1  OPN | -0.04  0.04 |

**(vii) Predictor:** PGK1, CA9

| **Notch signaling gene**  **(Dependent variable)** | **r** | **r^2^** | **p-value of regression model** | **β** | |
| --- | --- | --- | --- | --- | --- |
| Dll3 | 0.50 | 0.25 | 0.011 | PGK1  CA9 | 0.40  -0.01 |
| J1 | 0.49 | 0.24 | 0.013 | PGK1  CA9 | 1.12  0.12 |
| Hes1 | 0.49 | 0.24 | 0.012 | PGK1  CA9 | 0.42  -0.01 |
| Hes6 | 0.61 | 0.38 | 0.001 | PGK1  CA9 | 0.35  -0.008 |
| Hey1 | 0.42 | 0.18 | 0.042 | PGK1  CA9 | 0.14  -0.003 |

**(viii) Predictor:** VEGF, OPN

| **Notch signaling gene**  **(Dependent variable)** | **r** | **r^2^** | **p-value of regression model** | **β** | |
| --- | --- | --- | --- | --- | --- |
| Dll3 | 0.84 | 0.70 | 0.000 | VEGF  OPN | -0.05  0.21 |
| Hes6 | 0.51 | 0.26 | 0.009 | VEGF  OPN | 0.03  0.06 |
| Hey1 | 0.42 | 0.18 | 0.046 | VEGF  OPN | 0.004  0.036 |
| Hey2 | 0.58 | 0.34 | 0.001 | VEGF  OPN | -0.01  0.03 |

**(ix) Predictor:** VEGF, CA9

| **Notch signaling gene**  **(Dependent variable)** | **r** | **r^2^** | **p-value of regression model** | **β** | |
| --- | --- | --- | --- | --- | --- |
| J1 | 0.50 | 0.25 | 0.011 | VEGF  CA9 | 0.43  0.11 |
| Hes6 | 0.50 | 0.25 | 0.011 | VEGF  CA9 | 0.07  -0.006 |

**(x) Predictor:** OPN, CA9

| **Notch signaling gene**  **(Dependent variable)** | **r** | **r^2^** | **p-value of regression model** | **β** | |
| --- | --- | --- | --- | --- | --- |
| Dll3 | 0.83 | 0.68 | 0.000 | OPN  CA9 | 0.19  -0.005 |
| J1 | 0.48 | 0.23 | 0.016 | OPN  CA9 | 0.09  0.14 |
| Hes1 | 0.43 | 0.19 | 0.038 | OPN  CA9 | 0.11  -0.004 |
| Hes6 | 0.48 | 0.23 | 0.016 | OPN  CA9 | 0.08  -0.002 |
| Hey1 | 0.42 | 0.17 | 0.048 | OPN  CA9 | 0.04  0.00 |
| Hey2 | 0.57 | 0.32 | 0.002 | OPN  CA9 | 0.03  -0.001 |

**(xi) Predictor: HIF-1α, PGK1**, VEGF

| **Notch signaling gene**  **(Dependent variable)** | **r** | **r^2^** | **p-value of regression model** | **β** | |
| --- | --- | --- | --- | --- | --- |
| Notch4 | 0.46 | 0.22 | 0.054 | HIF-1α  PGK1  VEGF | 0.55  -0.11  0.03 |
| Dll3 | 0.64 | 0.41 | 0.001 | HIF-1α  PGK1  VEGF | 0.24  0.62  -0.16 |
| Hes6 | 0.55 | 0.30 | 0.010 | HIF-1α  PGK1  VEGF | 0.26  -0.008  0.04 |
| Hey1 | 0.52 | 0.27 | 0.020 | HIF-1α  PGK1  VEGF | 0.14  0.08  -0.01 |
| Hey2 | 0.47 | 0.22 | 0.050 | HIF-1α  PGK1  VEGF | 0.02  0.13  -0.03 |

**(xii) Predictor: HIF-1α, PGK1**, VEGF, CA9

| **Notch signaling gene**  **(Dependent variable)** | **r** | **r^2^** | **p-value of regression model** | **β** | |
| --- | --- | --- | --- | --- | --- |
| Dll3 | 0.74 | 0.54 | 0.000 | HIF-1α  PGK1  VEGF  CA9 | 0.11  0.98  -0.19  -0.01 |
| Hes1 | 0.55 | 0.30 | 0.025 | HIF-1α  PGK1  VEGF  CA9 | 0.27  0.34  -0.009  -0.01 |
| Hes6 | 0.65 | 0.42 | 0.002 | HIF-1α  PGK1  VEGF  CA9 | 0.17  0.23  0.02  -0.007 |
| Hey1 | 0.54 | 0.30 | 0.029 | HIF-1α  PGK1  VEGF  CA9 | 0.12  0.14  -0.02  -0.002 |
| Hey2 | 0.58 | 0.34 | 0.013 | HIF-1α  PGK1  VEGF  CA9 | -0.01  0.20  -0.04  -0.002 |

**(xiii) Predictor: HIF-1α, PGK1**, VEGF, OPN

| **Notch signaling gene**  **(Dependent variable)** | **r** | **r^2^** | **p-value of regression model** | **β** | |
| --- | --- | --- | --- | --- | --- |
| Dll3 | 0.89 | 0.79 | 0.000 | HIF-1α  PGK1  VEGF  OPN | -0.48  -0.07  -0.05  0.32 |
| Hes6 | 0.55 | 0.30 | 0.025 | HIF-1α  PGK1  VEGF  OPN | 0.24  -0.03  0.04  0.009 |
| Hey1 | 0.53 | 0.28 | 0.037 | HIF-1α  PGK1  VEGF  OPN | 0.19  0.13  -0.02  -0.02 |
| Hey2 | 0.64 | 0.41 | 0.002 | HIF-1α  PGK1  VEGF  OPN | -0.10  0.02  -0.02  0.05 |

Note: Only the Notch genes found to be significantly associated (p ≤ 0.05) with the predictor of regression model have been shown.

Abbreviations: r, correlation coefficient; r^2^, coefficient of determination; β, regression coefficient
