# Supplementary material for: A Combined Gene Signature of Hypoxia and Notch Pathway in Human Glioblastoma and Its Prognostic Relevance
Source: PLoS One. 2015 Mar 3;10(3):e0118201. doi: 10.1371/journal.pone.0118201 (PMC4348203; doi:10.1371/journal.pone.0118201)
Supplement: S12 Table — (DOC) [file pone.0118201.s018.doc]

**Table S12.** Survival time of 21 GBM patients with the corresponding mRNA expression of the identified components of hypoxia-Notch signaling axis

| Survival (days) | Sample ID | HIF-1α | PGK1 | VEGF | OPN | CA9 | Notch1 | Dll1 | Hes1 | Hes6 | Hey1 | Hey2 |
| --- | --- | --- | --- | --- | --- | --- | --- | --- | --- | --- | --- | --- |
| 1800 | GBM6 | 0.8 | **15.9** | **63.3** | **3.8** | **60.5** | 0.3 | 0.6 | 1.3 | 0.3 | 0.4 | 0.7 |
| 960 | GBM1 | 0.8 | 1.4 | 1.1 | 0.5 | **56.8** | 0.4 | **2.6** | 0.5 | 0.0 | 0.0 | 0.3 |
| 720 | GBM13 | 0.0 | **1.7** | 0.1 | 0.0 | **14.5** | 0.0 | 0.0 | 0.1 | 0.0 | 0.0 | 0.0 |
| 549+ | GBM31 | **3.0** | **10.9** | **125.7** | **2.9** | **537.2** | **7.2** | **35.4** | 0.8 | **6.8** | **10.0** | **1.6** |
| 397+ | GBM14 | **1.5** | **3.6** | 0.4 | 0.1 | **2.6** | 0.6 | **21.9** | 0.2 | **2.8** | **1.6** | 0.9 |
| 330 | GBM15 | 0.6 | 0.7 | **21.8** | 1.1 | **6.4** | 1.1 | **1.7** | 0.3 | 0.1 | 0.3 | 0.4 |
| 270 | GBM7 | **76.7** | **17.9** | **12.8** | **166.8** | 0.0 | 0.3 | **19.7** | **23.7** | **2.9** | **7.1** | 0.6 |
| 270 | GBM16 | **4.0** | **8.6** | **7.5** | **4.5** | **24.6** | **3.0** | **20.1** | 0.5 | 0.3 | 1.2 | 0.9 |
| 240 | GBM 28 | 0.0 | 1.3 | **61.9** | **2.4** | **101.6** | 0.2 | 1.3 | 0.4 | 0.0 | 0.1 | 0.1 |
| 170 | GBM9 | **10.6** | **3.2** | **7.9** | 1.3 | 0.3 | **3.7** | **2.0** | **9.8** | 0.0 | 0.9 | **4.3** |
| 102 | GBM25 | 1.0 | **1.7** | **1.6** | **8.8** | **87.5** | 0.0 | 0.2 | 0.1 | 0.0 | 0.4 | 0.0 |
| 44+ | GBM30 | 0.9 | **11.2** | **79.8** | **7.5** | **8.3** | **4.3** | **3.0** | **1.9** | **3.0** | 0.5 | 0.2 |
| 41+ | GBM26 | 0.0 | 0.1 | 0.3 | 0.1 | 1.1 | 0.0 | 0.0 | 0.0 | 0.0 | 0.0 | 0.0 |
| 30 | GBM 27 | **2.7** | **13.6** | **69.5** | **2.8** | **451.1** | **1.6** | **4.2** | **1.7** | 0.1 | **1.5** | 0.3 |
| 28 | GBM19 | **2.2** | **10.2** | **2.1** | **3.8** | 0.3 | **18.6** | **68.7** | **3.1** | **2.4** | **17.6** | 1.0 |
| 23 | GBM24 | **1.8** | **15.0** | **96.7** | **6.7** | **265.3** | 0.3 | **2.7** | 0.2 | 0.2 | **4.8** | 0.3 |
| 21 | GBM35 | **26.8** | **107.3** | **372.1** | **120.4** | **3118.4** | **2.0** | **52.1** | **2.9** | **3.2** | **5.0** | 1.0 |
| 16+ | GBM12 | 0.0 | 0.9 | **15.5** | 1.3 | **22.9** | 0.0 | 0.0 | 0.0 | 0.0 | 0.1 | 0.2 |
| 8 | GBM34 | 1.4 | **13.0** | **10.1** | **5.6** | **3.6** | 0.5 | **5.4** | **2.4** | 1.1 | 0.2 | 0.2 |
| 6 | GBM21 | **1.8** | **2.8** | **9.6** | **7.3** | **41.9** | 0.2 | 0.2 | 0.2 | 0.0 | 0.3 | 0.0 |
| 5 | GBM33 | **3.6** | **5.7** | **6.1** | **7.2** | **3.8** | **3.2** | **34.0** | 1.1 | **6.8** | **5.9** | **1.8** |

Note: ‘+’ denotes censored subjects. Gene expression values ≥1.5-fold have been marked in bold to indicate significant upregulation as compared to normal brain.
