# Supplementary material for: A Combined Gene Signature of Hypoxia and Notch Pathway in Human Glioblastoma and Its Prognostic Relevance
Source: PLoS One. 2015 Mar 3;10(3):e0118201. doi: 10.1371/journal.pone.0118201 (PMC4348203; doi:10.1371/journal.pone.0118201)
Supplement: S1 File — Supplementary Materials and Methods. (DOC) [file pone.0118201.s019.doc]

**Supplementary Data Text**

**Supplementary Materials and Methods**

**q-PCR and data analysis**

q-PCR was done in 10µl reaction volume containing 2.5µl cDNA template (5 times diluted reverse transcriptase reaction solution), 1µl 10x Syto9 (Invitrogen), 5 pmoles forward and reverse primer each, 0.5 U Taq DNA polymerase, 0.25µl of 10mM dNTP mix and 4.58µl nuclease free water. PCR cycling conditions were: Initial denaturation at 95ºC for 1 minute; 10 seconds at 95ºC, 20 seconds at annealing temperature and 30 seconds at 72ºC. The cycle was repeated 40 times followed by melt curve analysis. Fluorescence was acquired at 72ºC as well as at a temperature preceding the T_m_ of the PCR product. All reactions were performed in triplicates. Average C_T_ value of triplicate tubes was calculated for each gene.

PCR products were electrophoresed on 1.5% agarose gel followed by visualization on UV transilluminator and documentation using ChemiImager software in a gel documentation system (Alpha Innotech Corporation, USA).

When quantitation was done with respect to multiple reference genes (18s rRNA, POLR2A (RNA polymerase II) and PPIA (peptidylprolyl isomerase A); (Table S2)), Relative Expression Software Tool (REST (http://www.gene-quantification.de/rest.html)) was used. C_T_ values and amplification efficiency for each gene were taken from the Comparative Quantitation tool of RotorGene software. MIQE (Minimum Information for a qPCR Experiment) guidelines were followed while performing real-time PCR.

**Statistical analysis**

1. **Spearman’s rank correlation coefficient:** Spearman’s rank test (non-parametric analysis) was used for pairwise gene correlations at 1-tailed test of significance across 35 GBMs.
2. **Sensitivity and specificity analysis:** The predictive power of each hypoxia marker to identify increased expression of Notch genes in GBMs was calculated as sensitivity and specificity. Overexpression of a given Notch gene was taken to be the value greater than the median expression of the hypoxia marker in question. Therefore, in the binary classified dataset, the expression values of Notch genes were transformed as 0 or 1, 0 denoting values ≤ median expression of a hypoxia marker and 1 denoting values ≥ median expression of a hypoxia marker. The % sensitivity arose from those counts where both the hypoxia marker and Notch gene were equal to 1 whereas the % specificity result arose from the counts where both the hypoxia marker and Notch gene were equal to 0. Sensitivity/specificity ≥ 50% were taken as the cut-offs for estimating the diagnostic accuracy of a given predictor.
3. **Logistic regression:** The strength of association between each possible predictor (hypoxia marker) or combination of predictors (combination of hypoxia markers) and diagnosis of increased expression of Notch genes was evaluated using logistic regression. The hypoxia marker was assigned as an independent variable while each Notch gene was taken as a dependent variable. Linear regression analysis was done using *Enter method*. The different combinations of hypoxia markers that were assessed are as follows:
4. HIF-1α, PGK1
5. HIF-1α, VEGF
6. HIF-1α, OPN
7. HIF-1α, CA9
8. PGK1, VEGF
9. PGK1, OPN
10. PGK1, CA9
11. VEGF, OPN
12. VEGF, CA9
13. OPN, CA9
14. HIF-1α, PGK1, VEGF
15. HIF-1α, PGK1, VEGF, CA9
16. HIF-1α, PGK1, VEGF, OPN
17. HIF-1α, PGK1, VEGF, CA9, OPN

**Immunohistochemistry**

Thermo Scientific UltraVision Quanto Detection System HRP DAB kit was used as per manufacturer’s instructions with some modification. Primary antibodies used were: HIF-1α (polyclonal, 1:100, Sigma-Aldrich, USA) and VEGF (monoclonal, 1:100, BioSB). Staining without primary antibody in parallel served as negative control. Briefly, paraffin-embedded GBM and normal brain tissues were serially cut into 5µm thick sections which were dewaxed and rehydrated through an alcohol series. Antigen retrieval was done by boiling the sections in 0.1M citrate buffer (pH 6.0) and the endogenous peroxidase activities were blocked by incubation in 4% v/v H_2_O_2_. The sections were incubated in primary antibody followed by secondary antibody. After colour development, the sections were counterstained with hematoxylin followed by dehydration and permanent mounting.

**Western blot analysis**

Whole cell lysates from monolayer and gliomasphere cultures were prepared using triple detergent lysis buffer (50mM Tris-HCl (pH 8), 120mM NaCl, 1% NP-40, 0.1% SDS, 1% sodium deoxycholate, sodium azide and protease inhibitor cocktail (Sigma-Aldrich, USA)). Gliomaspheres were initially disintegrated using Stem Pro Accutase Cell Dissociation Reagent (Invitrogen, USA) followed by lysate preparation. 80µg of the lysates were loaded in 8%-15% SDS-PAGE gels. Resolved proteins in SDS-PAGE gel were transferred onto nitrocellulose membrane (MDI Membrane Technologies, USA). Blocking was done using 5% w/v Bovine Serum Albumin (BSA) in 1X TBS (pH 7.4) with 0.05% Tween-20 at room temperature for 2 hours. The blot was then incubated overnight with primary antibody at the appropriate dilution in 5% BSA in 1X TBS (pH 7.4) with 0.05% Tween-20. This was followed by incubation in the appropriate secondary antibody (anti-mouse or anti-rabbit-HRP conjugated; from Santa Cruz Biotechnology Inc., USA) diluted at 1:5000 in 5% BSA in 1X TBS (pH 7.4) with 0.05% Tween-20 for 1.5 hours at room temperature. The immunoreactive protein bands were detected using enhanced chemiluminescence Western blotting substrate (Pierce, Thermo Fisher Scientific, USA) as per manufacturer’s instructions followed by visualization and documentation in gel documentation system (Cell Biosciences, Inc., USA). Primary antibodies used were: HIF-1α (mouse monoclonal, 1:500, Novus Biologicals), CA9 (rabbit polyclonal, 1:2000, Abcam), PGK1 (mouse monoclonal, 1:1000, Santa Cruz Biotechnology Inc.), Notch-1 cleaved (rabbit polyclonal, 1:500, Abcam), Notch3 (rabbit polyclonal, 1:500, Santa Cruz Biotechnology Inc.), Hey1 (rabbit polyclonal, 1:500, Millipore), Hes1 (rabbit polyclonal, 1:500, Abcam), Sox2 (rabbit polyclonal, 1:1000, Santa Cruz Biotechnology Inc.) and β-actin (mouse monoclonal, 1:5000, Abcam).
