# Supplementary material for: A Combined Gene Signature of Hypoxia and Notch Pathway in Human Glioblastoma and Its Prognostic Relevance
Source: PLoS One. 2015 Mar 3;10(3):e0118201. doi: 10.1371/journal.pone.0118201 (PMC4348203; doi:10.1371/journal.pone.0118201)
Supplement: S2 File — . zip folder containing the R script master code and input data files for performing PCA and Kaplan-Meier survival analysis. (ZIP) [file pone.0118201.s020.zip › FILE S2- R ANALYSIS SUPPLEMENTAL CONTENT/master_code_July_30.doc]

**R script Master Code for performing PCA and KM analyses:**

**(To be copied and pasted in the console of R statistical software and executed by pressing ‘Ctrl+R’)**

\# The R code and associated data files should be used together with

\# the manuscript Irshad et al. 2014 titled "A combined gene signature of

\# hypoxia and Notch pathway in human glioblastoma and its

\# prognostic relevance"

\# Investigators: Khushboo Irshad, Subrata Sinha and Kunzang Chosdol

\# Analysis by: Saroj Kant Mohapatra and Khushboo Irshad

\# Date: Jul 30, 2014

###########################################################

\# Read the data

###########################################################

\# Change directory to wherever the data are stored

\# For the computer, use the following, or change appropriately

setwd("C:/R ANALYSIS SUPPLEMENTAL CONTENT")

dat <- read.table(file="genexp.txt", header=T, sep="\t", row.names=1)

\# Replace the zero-values with a small number that is half of the least

minval = min(dat[dat>0])

halfmin = minval/2

dat[dat==0] <- halfmin

\# log-transform the data

logdat <- log2(dat)

###########################################################

\# Beginning of Figure 2(i)

###########################################################

hif15 <- c("N1", "N2", "N3", "N4", "DLL1", "DLL3", "DLL4", "J1", "J2", "HES1", "HES2", "HES5", "HES6", "HEY1", "HEY2", "HIF1a")

seldat <- logdat[, hif15]

\# Principal Comonents Analysis

pcdat <- prcomp(t(seldat), center=T, scale.=TRUE)

pcres <- pcdat$rotation[,1:3]

mycols <- colorRampPalette(c("red","white","darkgreen"))(35)

par(mfrow=c(1,3))

plot(x=pcres[,1], y=pcres[,2], col=mycols, pch=16, cex=2,

xlab="PC1", ylab="PC2")

plot(x=pcres[,1], y=pcres[,3], col=mycols, pch=16, cex=2,

xlab="PC1", ylab="PC3")

plot(x=pcres[,2], y=pcres[,3], col=mycols, pch=16, cex=2,

xlab="PC2", ylab="PC3")

\# End of Figure 2(i)

##########################################################

\# Beginning of Figure 2(ii)

##########################################################

hif9 <- c("N1", "DLL1", "DLL4", "HES1", "HES2", "HES5", "HES6", "HEY1", "HEY2", "HIF1a")

seldat <- logdat[, hif9]

\# Principal Comonents Analysis

pcdat <- prcomp(t(seldat), center=T, scale.=TRUE)

pcres <- pcdat$rotation[,1:3]

mycols <- colorRampPalette(c("red","white","darkgreen"))(35)

par(mfrow=c(1,3))

plot(x=pcres[,1], y=pcres[,2], col=mycols, pch=16, cex=2,

xlab="PC1", ylab="PC2")

plot(x=pcres[,1], y=pcres[,3], col=mycols, pch=16, cex=2,

xlab="PC1", ylab="PC3")

plot(x=pcres[,2], y=pcres[,3], col=mycols, pch=16, cex=2,

xlab="PC2", ylab="PC3")

\# End of Figure 2(ii)

##########################################################

\# Beginning of Figure 2(iii)

##########################################################

hif7 <- c("N1", "DLL1", "HES1", "HES2", "HES6", "HEY1", "HEY2", "HIF1a")

seldat <- logdat[, hif7]

\# Principal Comonents Analysis

pcdat <- prcomp(t(seldat), center=T, scale.=TRUE)

pcres <- pcdat$rotation[,1:3]

mycols <- colorRampPalette(c("red","white","darkgreen"))(35)

par(mfrow=c(1,3))

plot(x=pcres[,1], y=pcres[,2], col=mycols, pch=16, cex=2,

xlab="PC1", ylab="PC2")

plot(x=pcres[,1], y=pcres[,3], col=mycols, pch=16, cex=2,

xlab="PC1", ylab="PC3")

plot(x=pcres[,2], y=pcres[,3], col=mycols, pch=16, cex=2,

xlab="PC2", ylab="PC3")

\# End of Figure 2(iii)

##########################################################

\# Beginning of Figure 3(i)

##########################################################

pgk15 <- c("N1", "N2", "N3", "N4", "DLL1", "DLL3", "DLL4", "J1", "J2", "HES1", "HES2", "HES5", "HES6", "HEY1", "HEY2", "PGK1")

seldat <- logdat[, pgk15]

\# Principal Comonents Analysis

pcdat <- prcomp(t(seldat), center=T, scale.=TRUE)

pcres <- pcdat$rotation[,1:3]

mycols <- colorRampPalette(c("red","white","darkgreen"))(35)

par(mfrow=c(1,3))

plot(x=pcres[,1], y=pcres[,2], col=mycols, pch=16, cex=2,

xlab="PC1", ylab="PC2")

plot(x=pcres[,1], y=pcres[,3], col=mycols, pch=16, cex=2,

xlab="PC1", ylab="PC3")

plot(x=pcres[,2], y=pcres[,3], col=mycols, pch=16, cex=2,

xlab="PC2", ylab="PC3")

\# End of Figure 3(i)

##########################################################

\# Beginning of Figure 3(ii)

##########################################################

pgk7 <- c("N1", "DLL1", "DLL4", "HES1", "HES6", "HEY1", "HEY2", "PGK1")

seldat <- logdat[, pgk7]

\# Principal Comonents Analysis

pcdat <- prcomp(t(seldat), center=T, scale.=TRUE)

pcres <- pcdat$rotation[,1:3]

mycols <- colorRampPalette(c("red","white","darkgreen"))(35)

par(mfrow=c(1,3))

plot(x=pcres[,1], y=pcres[,2], col=mycols, pch=16, cex=2,

xlab="PC1", ylab="PC2")

plot(x=pcres[,1], y=pcres[,3], col=mycols, pch=16, cex=2,

xlab="PC1", ylab="PC3")

plot(x=pcres[,2], y=pcres[,3], col=mycols, pch=16, cex=2,

xlab="PC2", ylab="PC3")

\# End of Figure 3(ii)

#########################################################

\# Beginning of Figure 3(iii)

#########################################################

pgk6 <- c("N1", "DLL1", "HES1", "HES6", "HEY1", "HEY2", "PGK1")

seldat <- logdat[, pgk6]

\# Principal Comonents Analysis

pcdat <- prcomp(t(seldat), center=T, scale.=TRUE)

pcres <- pcdat$rotation[,1:3]

mycols <- colorRampPalette(c("red","white","darkgreen"))(35)

par(mfrow=c(1,3))

plot(x=pcres[,1], y=pcres[,2], col=mycols, pch=16, cex=2,

xlab="PC1", ylab="PC2")

plot(x=pcres[,1], y=pcres[,3], col=mycols, pch=16, cex=2,

xlab="PC1", ylab="PC3")

plot(x=pcres[,2], y=pcres[,3], col=mycols, pch=16, cex=2,

xlab="PC2", ylab="PC3")

\# End of Figure 3(iii)

###########################################################

\# Beginning of Figure 4(i)

###########################################################

hif15 <- c("N1", "N2", "N3", "N4", "DLL1", "DLL3", "DLL4", "J1", "J2", "HES1", "HES2", "HES5", "HES6", "HEY1", "HEY2", "HIF1a")

seldat <- logdat[, hif15]

\# Principal Components Analysis

require("rgl")

pc <- princomp(logdat)

colour <- c(rep(7, nrow(pc$scores)))

names(colour) <- rownames(pc$scores)

upsmps <- paste("GBM", c(7,8,10,35,11,9,2,17,4,20,16,33), sep="")

dnsmps <- paste("GBM", c(1,32,15,29,5,22,26,3,12,13,23,28), sep="")

colour[upsmps] <- 2

colour[dnsmps] <- 3

colour <- as.numeric(colour)

plot3d(pc$scores[,1:3], col=colour, xlab="PC1", ylab="PC2", zlab="PC3", type="n")

text3d(x=pc$scores[,1], y=pc$scores[,2], z=pc$scores[,3],

texts=rownames(pc$scores), col=colour)

\# End of Figure 4(i)

#########################################################

\# Beginning of Figure 4(ii)

#########################################################

pgk15 <- c("N1", "N2", "N3", "N4", "DLL1", "DLL3", "DLL4", "J1", "J2", "HES1", "HES2", "HES5", "HES6", "HEY1", "HEY2", "PGK1")

seldat <- logdat[, pgk15]

\# Principal Components Analysis

require("rgl")

pc <- princomp(logdat)

colour <- c(rep(7, nrow(pc$scores)))

names(colour) <- rownames(pc$scores)

upsmps <- paste("GBM", c(7,8,10,35,11,9,2,17,4,20,16,33), sep="")

dnsmps <- paste("GBM", c(1,32,15,29,5,22,26,3,12,13,23,28), sep="")

colour[upsmps] <- 2

colour[dnsmps] <- 3

colour <- as.numeric(colour)

plot3d(pc$scores[,1:3], col=colour, xlab="PC1", ylab="PC2", zlab="PC3", type="n")

text3d(x=pc$scores[,1], y=pc$scores[,2], z=pc$scores[,3],

texts=rownames(pc$scores), col=colour)

\# End of Figure 4(ii)

#########################################################

\# Beginning of Figure 4(iii)

#########################################################

hif9 <- c("N1", "DLL1", "DLL4", "HES1", "HES2", "HES5", "HES6", "HEY1", "HEY2", "HIF1a")

seldat <- logdat[, hif9]

\# Principal Components Analysis

require("rgl")

pc <- princomp(logdat)

colour <- c(rep(7, nrow(pc$scores)))

names(colour) <- rownames(pc$scores)

upsmps <- paste("GBM", c(7,8,10,35,11,9,2,17,4,20,16,33), sep="")

dnsmps <- paste("GBM", c(1,32,15,29,5,22,26,3,12,13,23,28), sep="")

colour[upsmps] <- 2

colour[dnsmps] <- 3

colour <- as.numeric(colour)

plot3d(pc$scores[,1:3], col=colour, xlab="PC1", ylab="PC2", zlab="PC3", type="n")

text3d(x=pc$scores[,1], y=pc$scores[,2], z=pc$scores[,3],

texts=rownames(pc$scores), col=colour)

\# End of Figure 4(iii)

#########################################################

\# Beginning of Figure 4(iv)

#########################################################

pgk7 <- c("N1", "DLL1", "DLL4", "HES1", "HES6", "HEY1", "HEY2", "PGK1")

seldat <- logdat[, pgk7]

\# Principal Components Analysis

require("rgl")

pc <- princomp(logdat)

colour <- c(rep(7, nrow(pc$scores)))

names(colour) <- rownames(pc$scores)

upsmps <- paste("GBM", c(7,8,10,35,11,9,2,17,4,20,16,33), sep="")

dnsmps <- paste("GBM", c(1,32,15,29,5,22,26,3,12,13,23,28), sep="")

colour[upsmps] <- 2

colour[dnsmps] <- 3

colour <- as.numeric(colour)

plot3d(pc$scores[,1:3], col=colour, xlab="PC1", ylab="PC2", zlab="PC3", type="n")

text3d(x=pc$scores[,1], y=pc$scores[,2], z=pc$scores[,3],

texts=rownames(pc$scores), col=colour)

\# End of Figure 4(iv)

#########################################################

\# Beginning of Figure 4(v)

#########################################################

hif7 <- c("N1", "DLL1", "HES1", "HES2", "HES6", "HEY1", "HEY2", "HIF1a")

seldat <- logdat[, hif7]

\# Principal Components Analysis

require("rgl")

pc <- princomp(logdat)

colour <- c(rep(7, nrow(pc$scores)))

names(colour) <- rownames(pc$scores)

upsmps <- paste("GBM", c(7,8,10,35,11,9,2,17,4,20,16,33), sep="")

dnsmps <- paste("GBM", c(1,32,15,29,5,22,26,3,12,13,23,28), sep="")

colour[upsmps] <- 2

colour[dnsmps] <- 3

colour <- as.numeric(colour)

plot3d(pc$scores[,1:3], col=colour, xlab="PC1", ylab="PC2", zlab="PC3", type="n")

text3d(x=pc$scores[,1], y=pc$scores[,2], z=pc$scores[,3],

texts=rownames(pc$scores), col=colour)

\# End of Figure 4(v)

##########################################################

\# Beginning of Figure 4(vi)

##########################################################

pgk6 <- c("N1", "DLL1", "HES1", "HES6", "HEY1", "HEY2", "PGK1")

seldat <- logdat[, pgk6]

\# Principal Components Analysis

require("rgl")

pc <- princomp(logdat)

colour <- c(rep(7, nrow(pc$scores)))

names(colour) <- rownames(pc$scores)

upsmps <- paste("GBM", c(7,8,10,35,11,9,2,17,4,20,16,33), sep="")

dnsmps <- paste("GBM", c(1,32,15,29,5,22,26,3,12,13,23,28), sep="")

colour[upsmps] <- 2

colour[dnsmps] <- 3

colour <- as.numeric(colour)

plot3d(pc$scores[,1:3], col=colour, xlab="PC1", ylab="PC2", zlab="PC3", type="n")

text3d(x=pc$scores[,1], y=pc$scores[,2], z=pc$scores[,3],

texts=rownames(pc$scores), col=colour)

\# End of Figure 4(vi)

###########################################################

\# Beginning of Figure 5

###########################################################

\# setwd("C:/R ANALYSIS SUPPLEMENTAL CONTENT")

library(survival)

\# Set working directory

gbm <- read.delim(file="Table_9_with_censored_3rd.txt", header=TRUE, sep="\t")

mysurvplot <- function(cutoff=1.0, geneid="HIF1a") {

gbm$gstat <- as.integer(gbm[,geneid]>cutoff)

my.surv <- with(gbm, Surv(days,outcome))

my.fit <- survfit(my.surv~gstat, data=gbm)

plot(my.fit, col=c("darkgreen","red"), lwd=3,

xlab="Survival (days)", ylab="Survival (percent)",

main=paste("Survival analysis (",geneid," threshold = ", cutoff, ")", sep=""),

yscale=100)

survival_diff <- survdiff(my.surv~gstat, data=gbm)

print(survival_diff)

}

gname="HIF1a";cutoff=1.5

mysurvplot(cutoff=cutoff,geneid=gname)

\# p= 0.0205

scan()

gname="HES1";cutoff=1.5

mysurvplot(cutoff=cutoff,geneid=gname)

\# p= 0.0462

scan()

gname="OPN";cutoff=5.0

mysurvplot(cutoff=cutoff,geneid=gname)

\# p= 0.00172

scan()

\# Using multiple genes

gbm$gstat <- (with(gbm, as.integer(HIF1a+OPN+HES1>8.0)))

#gbm$gstat <- (with(gbm, as.integer(HIF1a>1.5 | OPN>5.0)))

my.surv <- with(gbm, Surv(days,outcome))

my.fit <- survfit(my.surv~gstat, data=gbm)

plot(my.fit, col=c("darkgreen","red"), lwd=3,

xlab="Survival (days)", ylab="Survival (percent)",

main="Survival analysis (HIF1a, OPN and HES1)",

yscale=100)

survdiff(my.surv~gstat, data=gbm)

\# p= 0.00081

\# End of Figure 5
